# Humans and Chimpanzees Display Opposite Patterns of Diversity in *Arylamine N-Acetyltransferase* Genes

**DOI:** 10.1534/g3.119.400223

**Published:** 2019-05-08

**Authors:** Christelle Vangenot, Pascal Gagneux, Natasja G. de Groot, Adrian Baumeyer, Médéric Mouterde, Brigitte Crouau-Roy, Pierre Darlu, Alicia Sanchez-Mazas, Audrey Sabbagh, Estella S. Poloni

**Affiliations:** * Department of Genetics and Evolution, Anthropology Unit, University of Geneva, Switzerland; † Departments of Pathology and Anthropology, CARTA (Center for Academic Research and Training in Anthropogeny), University of California San Diego, La Jolla; ‡ Department of Comparative Genetics and Refinement, Biomedical Primate Research Centre, the Netherlands; § Zoologischer Garten Basel AG, Basel, Switzerland; ** CNRS, Université Toulouse 3 UPS, ENFA, UMR 5174, Toulouse, France; †† CNRS/Muséum National d’Histoire Naturelle, UMR 7206 Paris, France; ‡‡ Institute of Genetics and Genomics in Geneva (IGE3), Switzerland; §§ UMR 216 MERIT, IRD, Université Paris Descartes, Sorbonne Paris Cité, Paris, France

**Keywords:** Arylamine N-acetyltransferases, multigenic family, drug metabolism, great apes, natural selection

## Abstract

Among the many genes involved in the metabolism of therapeutic drugs, human arylamine *N*-acetyltransferases (*NATs*) genes have been extensively studied, due to their medical importance both in pharmacogenetics and disease epidemiology. One member of this small gene family, *NAT2*, is established as the locus of the classic human acetylation polymorphism in drug metabolism. Current hypotheses hold that selective processes favoring haplotypes conferring lower *NAT2* activity have been operating in modern humans’ recent history as an adaptation to local chemical and dietary environments. To shed new light on such hypotheses, we investigated the genetic diversity of the three members of the *NAT* gene family in seven hominid species, including modern humans, Neanderthals and Denisovans. Little polymorphism sharing was found among hominids, yet all species displayed high *NAT* diversity, but distributed in an opposite fashion in chimpanzees and bonobos (*Pan* genus) compared to modern humans, with higher diversity in *Pan* species at *NAT1* and lower at *NAT2*, while the reverse is observed in humans. This pattern was also reflected in the results returned by selective neutrality tests, which suggest, in agreement with the predicted functional impact of mutations detected in non-human primates, stronger directional selection, presumably purifying selection, at *NAT1* in modern humans, and at *NAT2* in chimpanzees. Overall, the results point to the evolution of divergent functions of these highly homologous genes in the different primate species, possibly related to their specific chemical/dietary environment (exposome) and we hypothesize that this is likely linked to the emergence of controlled fire use in the human lineage.

Hominid species, the so-called “great apes”, share a recent common history that makes the genomic diversity of our closest relatives highly informative in evolutionary studies on our own species and more broadly on all great apes. This potential to answer evolutionary questions regarding all hominid species, including our own, is extensively exploited, and notably so in the last half decade thanks to the accelerated pace at which whole genome sequences are generated (Kuhlwilm *et al.* 2016). As foreseen by Olson and Varki (2003), analyzing genetic and genomic diversity in the great apes is also providing novel insights of medical interest (*e.g.*, (Enard *et al.* 2002; Bergfeld *et al.* 2017; Solis-Moruno *et al.* 2017). Indeed, physiological differences between species, whether they emerged through demographic processes or as adaptive responses might offer insights into present-day questions regarding human health and disease (Olson and Varki 2003; O’Bleness *et al.* 2012).

*Arylamine N-acetyltransferases* (*NATs*) genes are members of a small multigene family coding for enzymes that biotransform numerous compounds. In humans, its two functional members, *NAT1* and *NAT2*, show differences in expression patterns and substrate specificity, while the third member (*NATP*) is a pseudogene. While the NAT2 isoenzyme has a major role in the metabolism of xenobiotics, including therapeutic drugs and carcinogens, growing evidence supports an additional role for NAT1 in physiological processes (notably folate and methionine metabolism) and cancer cell biology. The three *NAT* genes reside in a 200 Kb region on the short arm of chromosome 8, and each of the two functional genes has a single, uninterrupted, 870 bp-long coding exon that produces a protein of 290 amino acids (Blömeke and Lichter 2018; Sim and Laurieri 2018). Phylogenetic analyses of *NAT* sequences point to multiple episodes of *NAT* gene duplication or gene loss during vertebrate evolution (Sabbagh *et al.* 2013). However, in Simiiformes (monkeys and apes, including humans) the evidence suggests the occurrence of a single duplication event prior to their divergence, leading to the *NAT1* and *NAT2* paralogs, whereas a subsequent duplication of *NAT2*, which probably occurred in the common ancestor to Catarrhini (African and Eurasian monkeys and apes, including humans), gave rise to the *NATP* pseudogene. Nucleotide sequence identity between the three *NAT* paralogs in humans is high: there is 81% homology between *NAT1* and *NAT2* coding exons, and 79% with the *NATP* pseudogene, while protein sequence identity between the two NAT enzymes is at 87% (Sabbagh *et al.* 2013). Homology between orthologous nucleotide sequences of humans, chimpanzees and gorillas is also high, about 98–99% for *NAT1* and *NAT2*, and 96–97% for *NATP*, according to the human (GRCh37/hg19, (Lander *et al.* 2001)), chimpanzee (panTro4, (The Chimpanzee Sequencing and Analysis Consortium 2005)) and gorilla (gorGor4, (Scally *et al.* 2012)) reference genomes, with no clear distinction between these three great ape species. Indeed, identity at *NAT1* between the human and chimpanzee reference sequences is 98.5%, compared to 99% between human and gorilla, and 98.5% between chimpanzee and gorilla; at *NAT2* it is 98.6% between human and chimpanzee, 98.8% between human and gorilla, and 98.9% between chimpanzee and gorilla; and at *NATP* it is 96.8% between human and chimpanzee, 95.8% between human and gorilla, and 96.1% between chimpanzee and gorilla. These occurrences of apparent incomplete lineage sorting (although based on very slight differences) are not surprising since it is estimated that about a third of the gorilla genome is more similar to that of humans or chimpanzees than the human and chimpanzee genomes are to each other (Scally *et al.* 2012; Kronenberg *et al.* 2018). It highlights the complex speciation history of great apes, which possibly included several admixture events, such as those recently evidenced between bonobos and the ancestors of Central and Eastern chimpanzees (de Manuel *et al.* 2016).

Due to their involvement in inter-individual variation in response to therapeutic treatments, the molecular diversity of *NAT* genes in human populations has been intensively studied (Sabbagh *et al.* 2018). In particular, the *NAT2* encoded enzyme, mainly expressed in the liver, small intestine and colon, is involved in the metabolic breakdown of several clinically relevant compounds (Hein 2002), including isoniazid, a first-line antibiotic included in anti-tuberculosis therapies since the 1950s (Zumla *et al.* 2013). Yet, it is safe to assume that none of these compounds have had any evolutionary impact on human *NAT2* evolution. The single coding exon of *NAT2* is highly polymorphic in humans, and it has been shown that the efficacy and/or toxicity of several clinically important drugs are associated with variation in enzymatic activity conferred by different *NAT2* variants (Meyer 2004; Agundez 2008a; McDonagh *et al.* 2014). This, together with the involvement of the enzyme in the detoxification (N, O or N,O-acetylation) of numerous carcinogens, motivated numerous studies on the evolution of the diversity of *NAT2* in human populations. Current hypotheses hold that geographically- or culturally-restricted selective processes favoring one or several haplotypes conferring lower NAT2 activity (as adaptations to specific chemical environments or dietary habits) have been operating in the recent history of human populations (Patin *et al.* 2006a; Patin *et al.* 2006b; Fuselli *et al.* 2007; Luca *et al.* 2008; Magalon *et al.* 2008; Sabbagh *et al.* 2008; Mortensen *et al.* 2011; Sabbagh *et al.* 2011; Patillon *et al.* 2014; Podgorná *et al.* 2015; Valente *et al.* 2015; Bisso-Machado *et al.* 2016); see also (Sabbagh *et al.* 2018) for a review). Interestingly, the discovery of a strong association of decreased insulin sensitivity with a non-synonymous polymorphism in *NAT2* (Knowles *et al.* 2016) brings indirect support to the hypothesis of dietary-linked selective pressures exerted on the evolution of this gene. *NAT1* is also involved in N-acetylation reactions of numerous compounds, and it is mainly expressed in the liver. Moreover, as shown in the AceView browser (Thierry-Mieg and Thierry-Mieg 2006), it is also expressed in kidneys, lung, and blood cells, particularly in humans, and less so in chimpanzees. However, in contrast to *NAT2*, *NAT1* is substantially less polymorphic in humans, and it is currently held that the accumulation of molecular variation in the *NAT1* coding exon, in particular non-synonymous variation, has been hampered by relatively strong purifying selection acting on the gene (Patin *et al.* 2006a; Mortensen *et al.* 2011; Sabbagh *et al.* 2018). *NATP*, the third identified member of this family of genes in the human genome bears several loss-of-function mutations and transcriptome studies indicate that it is barely if ever transcribed (as shown in the EBI Gene Expression Atlas, entry ENSG00000253937 at www.ebi.ac.uk/gxa/home).

One approach to examine hypotheses of recent adaptation in acetylation activity in humans is to investigate the diversity of *NAT* genes in humans’ closest relatives. To this aim, we Sanger sequenced the three members of the *NAT* gene family in 84 great ape DNA samples, including 68 chimpanzees of the *Pan troglodytes verus* sub-species (Western chimpanzees). We completed this dataset with 231 *NAT* sequence genotypes produced with NextGen technology by the Great Ape Genome Project (GAGP) (Prado-Martinez *et al.* 2013). We also examined *NAT* sequence variation in reference genomes of ancient hominins (*i.e.*, *Homo sapiens* from Ust’-Ishim, Neanderthal and Denisova). Similarly to humans, we found high levels of nucleotide and haplotype diversity in non-human primates, but distributed differently in *Pan*, such that diversity is higher at *NAT1* and lower at *NAT2*, whereas the opposite is observed in humans. Hence we hypothesize that the highly homologous *NAT1* and *NAT2* genes evolved some divergence in functionality between species in the course of hominid history, and we discuss this hypothesis in relation to changes in the chemical or dietary environment, *i.e.*, the exposome (Wild 2012) in which humans and chimpanzees have evolved.

## Materials and Methods

### Great ape DNA samples

Eighty-four DNA samples of great apes were analyzed in this study. These comprised DNA from 68 *Pan troglodytes verus* (Western chimpanzee) individuals, one *P. t. troglodytes* (Central chimpanzee) female, one *P. t. schweinfurthii* (Eastern chimpanzee) female, one *P. paniscus* (bonobo) male, five *Gorilla gorilla* and eight *Pongo abelii* (Sumatran orangutan) individuals. Among the 68 *P. t. verus* samples, 40 were from members of the Biomedical Primate Research Centre (BPRC) colony, 26 from the Center for Academic Research and Training in Anthropogeny (CARTA) and two from the Basel zoo (Supplementary Figure S1). These samples and their corresponding collections are described in Supplementary File S1.

### Sanger sequencing of NAT genes

We sequenced the three segments in the *NAT* region that include the single coding exons of *NAT1* and *NAT2* and the homologous DNA stretch of *NATP* in the 84 DNA samples of great apes available for this study. Supplementary Table S1 lists the primers used in this study both for PCR amplification and forward and reverse sequencing of each of the three *NAT* loci in great apes. PCR conditions are provided in Supplementary File S1. Sanger sequencing was outsourced to Retrogen (San Diego, California, USA) and Macrogen (Seoul, South Korea). Supplementary Table S2 lists the available information on all non-human samples considered in this study, including those retrieved from the GAGP (see below).

### Alignment of NAT sequences

All *NAT* sequences obtained were aligned on the homologs from the reference or draft reference assemblies of *Homo sapiens* (GRCh37/hg19, February 2009), *Pan troglodytes verus* (panTro4, February 2011), *Pan paniscus* (panPan1, May 2012), *Gorilla gorilla gorilla* (gorGor4, December 2014, as well as gorGor3 for verification, since these two reference sequences are not identical) and *Pongo pygmaeus abelii* (ponAbe2, July 2007), respectively, all downloaded from the UCSC Genome Browser (Kent *et al.* 2002, genome.ucsc.edu). Alignment was performed blind of the known relationships between individuals.

### Retrieval of NAT sequences or polymorphic positions from public repositories

For comparison purposes, we retrieved *NAT* genotypes from the published unphased genomes of 79 great apes generated in the GAGP by NGS (Prado-Martinez *et al.* 2013), namely from 25 chimpanzees (including a hybrid *P. t. verus/troglodytes* individual), 13 bonobos, 31 gorillas and 10 orangutans. Two individuals, Harriet (*P. t. schweinfurthii*) and Boscoe (*P. t. verus*, Supplementary Figure S1), were in common in the GAGP and CARTA datasets, thus allowing a control of Sanger and NGS sequencing results. We extracted the relevant part of the available VCF files. Further details are provided in Supplementary File S1. All detected polymorphic positions in the Sanger sequenced samples of this study and retrieved from the GAGP VCF files are detailed in Supplementary Tables S3, S4 and S5, for *NAT1*, *NAT2* and *NATP*, respectively.

To allow comparison of segregating sites at the *NAT* genes among all hominids (*i.e.*, all great apes, including humans), we recorded all human polymorphisms reported by the consensus gene nomenclature of human *NAT* alleles (http://nat.mbg.duth.gr/, accessed in August 2015), complemented with haplotype data from 1000 Genomes Phase 1 (The Genomes Project Consortium 2012) and published data (Patin *et al.* 2006a; Sabbagh *et al.* 2008; Mortensen *et al.* 2011; Podgorná *et al.* 2015). These are also reported in Supplementary Tables S3, S4 and S5.

### Inference of NAT haplotypes in the genus Pan

Diploid haplotypes were inferred for all *Pan* individuals (but not for the other great ape samples, see Supplementary File S1) using PHASE version 2.1.1 (Stephens *et al.* 2001; Stephens and Scheet 2005). The program uses a Bayesian statistical method based on an approximate coalescent model for reconstructing haplotypes from genotype data. PHASE implements a recombination method (the –MR option), which allows specifying the relative physical location of each SNP and accounts for the decay in linkage disequilibrium with distance.

### Constitution of two samples of Pan troglodytes verus unrelated individuals

We considered separately two samples of unrelated individuals from the Western (*P. t. verus*) chimpanzee sub-species, BPRC and San Diego (Supplementary File S1), notably due to significant differentiation between these two samples at the *NATP* pseudogene (Supplementary Table S6, see results).

### NAT haplotypes in humans

A dataset of *NAT* haplotypes’ frequency distributions in human population samples was assembled with published *NAT* sequences of same length as those of *Pan*, obtained through a comprehensive literature search at the time of the study. Only populations represented by samples including at least 15 individuals (30 chromosomes) were considered. We thus used published data samples from Mortensen *et al.* (2011) and from Sabbagh *et al.* (2008). We also extracted *NAT1*, *NAT2* and *NATP* phased genotypes from the 1000 Genomes Phase 1 dataset (The Genomes Project Consortium 2012), see Supplementary File S1. In total, the human dataset consists of 20, 18 and 18 samples of unrelated individuals, from human populations distributed on four continents (Sub-Saharan Africa, Europe, East Asia and America), for *NAT1*, *NAT2* and *NATP*, respectively (Supplementary Table S7).

### NAT polymorphisms in ancient genomes of hominins

Variant calls in the homologous *NAT* sequences of ancient genomes of the genus *Homo*, namely Neanderthal, Altai and the composite genome of three individuals from Vindija, (Green *et al.* 2010; Prüfer *et al.* 2014), Denisova (Meyer *et al.* 2012), and those from the most ancient modern human sequenced genome, Ust’-Ishim (Fu *et al.* 2014), were examined in both the UCSC Genome Browser (https://genome.ucsc.edu/) and the ancient genome browser at the Max Planck Institute for Evolutionary Anthropology (http://www.eva.mpg.de/neandertal/index.html), the latter including the recently published high-coverage Vindija Neanderthal genome (Prüfer *et al.* 2017). For each of the two functional genes, we screened 2 Kb of the reference human genome sequence (Hg19/GRCh37) encompassing the coding exon (positions 18’079’000 to 18’081’000 for *NAT1*, and 18’257’000 to 18’259’000 for *NAT2*). For the *NATP* pseudogene, we screened 2 Kb of homologous sequence (18’227’600 to 18’229’600).

### Analysis of diversity of NAT genes in the genus Pan and comparison with humans and other great apes

Frequency distributions of *NAT* haplotypes in the *Pan* and human population samples were used to test for possible deviations from Hardy Weinberg equilibrium, to estimate expected heterozygosity, (*h*, equivalent to Nei’s gene diversity, (Nei 1987)) and nucleotide diversity (*π*), to estimate levels of population differentiation (Φ_ST_ statistics), and to test for possible departure from selective neutrality and demographic equilibrium (Ewens-Watterson homozygosity test, Tajima’s *D* test and Fu’s *F*_S_ test,), with the program Arlequin ver. 3.5 (Excoffier and Lischer 2010). Each of these three latter tests relies on different summaries of diversity (homozygosity for the Ewens-Watterson test, number of polymorphic sites and nucleotide diversity for Tajima’s *D*, and number of different haplotypes and nucleotide diversity for Fu’s *F*_s_), and only Tajima’s *D* and Fu’s *F*_s_ tests explicitly account for the mutational events distinguishing haplotypes. Statistical significance was assessed by generating 100’000 random samples under the null conditions of no selection and constant population size. For all tests that revealed at least one significant departure from the null hypothesis in one population (species, sub-species, or collection in the case of *P. t. verus*), the Holm correction method implemented in R (R Core Team 2013) was applied to control for type I error rate (Holm 1979), so as to obtain adjusted *P*-values. Arlequin was also used to infer population pairwise Φ_ST_ values (between species, sub-species, or collections) under the AMOVA framework, and their statistical significance was assessed with 100’000 permutations. The parameters used to estimate Φ_ST_ values were obtained with MEGA ver. 7 (Kumar *et al.* 2016). These parameters are: the molecular model (Tamura’s distance for all three *NAT* loci), the gamma parameter (no gamma correction for *NAT1* and *NAT2*, gamma = 0.05 for *NATP*) and the transition to transversion ratio (2.67, 2.0 and 2.5 for *NAT1*, *NAT2* and *NATP*, respectively). The program Network ver. 5.0 (Bandelt *et al.* 1999) was used to construct median-joining networks of *NAT* haplotypes in *Pan*.

### Prediction of functional impact of specific mutations in Pan haplotypes at the NAT1 and NAT2 loci

Phenotypic predictions of the functional impact of specific mutations in *Pan NAT1* and *NAT2* haplotypes were performed with three online software tools (analysis done in May 2017): PolyPhen (Adzhubei *et al.* 2010), SIFT (Sim *et al.* 2012) and the PANTHER cSNP Scoring tool (Tang and Thomas 2016). These three tools are able to predict the effect of a single nonsynonymous substitution on a protein sequence (Supplementary File S1). To investigate and compare the results returned by these methods, we first applied the three tools on human haplotypes of known phenotypes (Supplementary File S1). For the analysis of *Pan* haplotypes, we ran all three prediction tools with the default options, searching the UniProtKB/TrEMBL protein database (release 2010_09) with SIFT, and specifying *Pan troglodytes* as reference organism in PANTHER cSNP Scoring.

### Data availability

File S1 contains detailed descriptions of the protocols used for DNA amplification, PCR product purification and sequencing of great ape samples, retrieval of unphased *NAT* polymorphic positions from the Great Ape Genome Project (GAGP), inference of Pan *NAT* haplotypes, and retrieval of phased human *NAT* haplotypes from the 1000 Genomes Project. Table S1 lists the PCR and sequencing primers used for Sanger sequencing of *NAT* genes in non-human great ape DNA samples, and available information on these samples is provided in Table S2. Hominid polymorphic positions detected in *NAT1*, *NAT2*, and *NATP* are listed in Tables S3, S4, and S5, respectively. Table S6 reports estimates of differentiation levels between *Pan* (sub-)species. Table S7 lists the human population samples included in the modern human dataset, with associated diversity estimates and results of Hardy-Weinberg equilibrium tests. Supplementary information on results is also provided in File S1. All supplementary material has been uploaded to figshare. The 247 *NAT* sequenced genotypes obtained in this study are available in GenBank with accession numbers MK244999-MK245288 and MK245291-MK245459. Supplemental material available at FigShare: https://doi.org/10.25387/g3.7928192.

## Results

In this study, we Sanger sequenced approximately 1 Kb of homologous sequence in each of the three members of the *arylamine N-acetyltransferase* (*NAT*) gene family, *NAT1*, *NAT2* and the *NATP* pseudogene, in 84 great ape samples, of which 68 are chimpanzees of the *Pan troglodytes verus* sub-species. Out of the 84 DNA samples of great apes available, we obtained 248 *NAT* genotypes (83, 81 and 83 *NAT1*, *NAT2* and *NATP* genotypes, respectively, Supplementary File S1). We extended our dataset with 231 *NAT* genotypes from 79 individuals belonging to six great ape (sub-)species retrieved from the GAGP (Prado-Martinez *et al.* 2013). As reported in Tables 1 and 2, the total dataset assembled for analysis thus included 93 genotypes from four *Pan troglodytes* (common chimpanzee) sub-species for *NAT1* and *NAT2* (96 for *NATP*), 14 genotypes from *Pan paniscus* (bonobo) for each of the 3 *NAT* genes, 35 genotypes from two *Gorilla* species for *NAT2* and *NATP* (36 for *NAT1*), and 17 genotypes from two *Pongo* (orangutan) species for *NAT1* and *NAT2* (18 for *NATP*). For comparative analysis purposes, we also assembled a human *NAT* dataset totalizing 1’159 to 1’240 unrelated individuals from 18 to 20 populations distributed on four continents.

**Table 1:** **Segregating sites identified in the three *NAT* gene paralogs shared among different (sub-)species of hominids***^a^*.

Gene				*Homo*					*Gorilla**^c^*		*Pongo**^d^*		*Pan**^e^*				
					Ancient genomes												
Position in human reference sequence*^b^*	Human cds	SNP rs identifier	Alleles (amino acid change if non-synonymous)	*Homo sapiens*	*Ust’Ishim*	*Altai*	*Vindija*	*Denisova*	*Gorilla gorilla*	*Gorilla beringei*	*Pongo abelii*	*Pongo pygmaeus*	*P. t. verus*	*P. t. ellioti*	*P. t. troglodytes*	*P. t. schweinfurthii*	*Pan paniscus*
***NAT1***																	
Total sample size*^f^*									33	3	12	5	72	10	5	6	14
**18080001**	**445**	rs4987076	**A/G (I149V)**														
18080015	459	rs4986990	G/A														
**18080196**	**640**	rs4986783	**G/T (A214S)**														
***NAT2***																	
Total sample size*^f^*									32	3	12	5	72	10	5	6	14
**18257704**	**191**	rs1801279	**G/A (R64Q)**						T	T							
18257795	282	rs1041983	C/T														
18257858	345	rs45532639	C/T										A	A	A	A	A
**18258019**	**506**	rs200585149	**A/G (Y169C)**														
**18258091**	**578**	rs79050330	**C/T (T193M)**														
***NATP***																	
Total sample size*^f^*									32	3	13	5	75	10	5	6	14
18228246		rs73590295	T/C														
18228285			T/A														
18228458		rs372738250	G/A														
18228616		rs35548819	T/C														
18228661		rs546009408	G/A														
18228673		rs115350875	T/C														
18228727		rs530022558	G/A														
18228959			T/C														
18229104		rs74444655	T/C														

a Boxes shaded in light gray indicate the presence of the polymorphism in the relevant species/sub-species and those shaded in dark gray indicate fixation (detection for ancient genomes) of the derived allele in the species (if different from the human derived allele, the allele is indicated in the box).

b The screened segments for the *NAT1*, *NAT2* and *NATP* homologous sequences span from 18’079’545 to 18’080’447 (903 bp including the *NAT1* coding exon), 18’257’489 to 18’258’603 (1,115 bp including the *NAT2* coding exon) and 18’228’116 to 18’229’117 (1,002 bp including the *NATP* pseudogene) respectively, on chromosome 8 in the human reference sequence GRCh37/hg19. Non-synonymous mutations are shown in bold type.

c Based on the individuals of this study, the gorillas of Prado-Martinez *et al.* (2013) and the *Gorilla gorilla gorilla* draft assembly reference sequence (gorGor4, December 2014).

d Based on the individuals of this study, the orangutans of Prado-Martinez *et al.* (2013) and the *Pongo pygmaeus abelii* draft assembly reference sequence (ponAbe2, July 2007).

e Polymorphism recording is based on the chimpanzee and bonobo individuals of the present study and those of Prado-Martinez *et al.* (2013), cross-checked with the *Pan troglodytes verus* assembly reference sequence (panTro4, February 2011) and the *Pan paniscus* draft assembly reference sequence (panPan1, May 2012).

f Total number of genotypes, including genotypes of individuals deduced from their descendants (see Supplementary Figure S1A and Supplementary File S1).

**Table 2:** **Segregating sites identified in the three *NAT* gene paralogs in the different (sub-)species of the genus *Pan*, with paralogous positions in humans shown in the three last columns***^a^*.

Gene		*Pan* species/sub-species								
Position in human reference sequence*^b^*	Alleles (amino acid change if non-synonymous)	*P. troglodytes* (common chimpanzee)*^c^*					*P. paniscus* (bonobo)*^e^*	*Homo sapiens*		
		*P. t. verus* (Western)	*P. t. ellioti* (Nigeria-Cameroon)	*P. t. troglodytes* (Central)	*P. t. schweinfurthii* (Eastern)	Hybrid*^d^*		Human cds	Fixed position	Variable position (SNP rs identifier in Ensembl)*^f^*
***NAT1***										
*Pan* total sample size*^g^*		72	10	5	6	1	14			
**18079632**	**G/A (D26N)**							**76**	**G**	
18079703	C/T							147	C	
18079859	C/T							303	C	
**18079897**	**T/C (I114T)**							**341**		**T/A/C (rs145975713)**
18079925	T/C							369	T	
18080014	C/T							458		C/T (rs374226986)
**18080074**	**A/C (E173A)**							**518**	**A**	
**18080153**	**T/G (I199M)**							**597**	**T**	
**18080316**	**G/C (E254Q)**							**760**	**G**	
**18080345**	**A/G (I263M)**							**789**	**A**	
***NAT2***										
*Pan* total sample size*^g^*		72	10	5	6	1	14			
18257549	T/C							36	T	
**18257585**	**A/C (L24F)**							**72**	**A**	
**18257658**	**G/A (E49K)**							**145**	**G**	
**18257704**	**G/A (R64Q)**							**191**		**G/A (rs1801279)**
**18258027**	**A/G (N172D)**							**514**	**A**	
**18258091**	**C/T (T193M)**							**578**		**C/T (rs79050330)**
18258302	G/T							789	T	
18258447	G/A							934*^h^*	G	
18258462	C/G							949*^h^*	C	
***NATP***										
*Pan* total sample size*^g^*		75	10	5	6	1	14			
18228146	G/T							—	G	
18228189	C/T							—	C	
18228238	A/G							—	A	
18228242	A/G							—	A	
18228285*^i^*	T/A		N		N			—		T/A
18228304	C/T							—	C	
18228368	T/C							—	T	
18228404	C/T							—	C	
18228501	C/T							—	C	
18228543	A/G							—	A	
18228560	C/A							—	C	
18228582	G/T							—	G	
18228614	G/T							—	G	
18228659	G/A							—	G	
18228660	C/T							—	G	
18228661	G/A							—		G/A (rs546009408)
18228748	C/T							—	T	
18228771	C/T							—	T	
18228959	T/C							—	T	
18229057	G/A							—	C	
18229103	A/T							—	A	

a Boxes shaded in light gray indicate the presence of the polymorphism in the relevant *Pan* species/sub-species and those shaded in dark gray indicate a fixation of the derived allele in the Bonobo species.

b The screened segments for the *NAT1*, *NAT2* and *NATP* homologous sequences span from 18’079’545 to 18’080’447 (903 bp including the *NAT1* coding exon), 18’257’489 to 18’258’603 (1,115 bp including the *NAT2* coding exon) and 18’228’116 to 18’229’117 (1,002 bp including the *NATP* pseudogene) respectively, on chromosome 8 in the human reference sequence GRCh37/hg19. Non-synonymous mutations are shown in bold type.

c Polymorphism recording is based on the individuals of the present study and the chimpanzees of Prado-Martinez *et al.* (2013) cross-checked with the *Pan troglodytes verus* assembly reference sequence (panTro4, February 2011).

d Hybrid Western (*P. t. verus*) / Central (*P. t. troglodytes*) individual.

e Based on the individual of this study (Bonobo), the bonobos of Prado-Martinez *et al.* (2013) and the *Pan paniscus* draft assembly reference sequence (panPan1, May 2012).

f With the exception of human *NAT2* polymorphisms rs1801279 (G/A at cds position 191) and rs79050330 (C/T at cds position 578), which are common variants in humans, all other human polymorphisms, including those with a SNP rs identifier, are rare variants, detected with a highest population MAF < 0.01 in Ensembl (http://www.ensembl.org/Homo_sapiens/Info/Index).

g Total number of genotypes, including genotypes of individuals deduced from their descendants (see Supplementary Figure S1A and Supplementary File S1).

h Non-coding positions downstream of *NAT2* coding exon (3′UTR region).

i Undefined position in some Nigeria-Cameroun and Eastern chimpanzee samples (indicated by N, see Supplementary Table S5). The human T/A SNP at position 18’228’285, at present only described in Mortensen *et al.* (2011) at very low frequency, is not reported in Ensembl; we note that Ensembl reports another rare T/A SNP, with associated SNP identifier rs546046491, in the next contiguous position (18’228’286), and both variants are embedded in a stretch of T nucleotides on the human reference sequence (from 18’228’278 to 18’228’286).

In spite of the high level of known homology between the three *NAT* genes, approximately 8% nucleotide positions in *NAT1* (76 out of 903 bp), 10% in *NAT2* (117 out of 1’115 bp), and 15% in *NATP* (151 out of 1’002 bp) are segregating positions in hominids, that were found to either be divergent between species, polymorphic within a species, or both (Supplementary Tables S3, S4, and S5). Among them, 21 (28%) substitutions in *NAT1*, 38 (32%) in *NAT2*, and 77 (51%) in *NATP* correspond to inter-species divergence.

### NAT polymorphisms in hominids and polymorphism sharing among species

Despite the high numbers of segregating sites detected at the three *NAT* genes in hominids, little polymorphism was found to be shared between humans, gorillas, orangutans and *Pan*: two SNPs at *NAT1*, four at *NAT2* and six at the *NATP* pseudogene (Table 1).

No polymorphic position was found shared between the genus *Pan* and the other great ape species at the *NAT1* gene (Table 1 and Supplementary Table S3). Nonetheless, the proportion of non-synonymous SNPs in *Pan* (60%) is similar to that observed in humans (59%) and orangutans (56%), while it is lower in gorillas (25%).

Humans and orangutans were found to share two *NAT1* SNPs, *i.e.*, non-synonymous A/G at human cds position 445 (rs4987076) and synonymous G/A at 459 (rs4986990), the former being also observed in gorillas. These two polymorphisms, found at low frequencies in humans today, along with a third one not observed in gorillas or orangutans (non-synonymous 640 G/T, rs4986783), were nevertheless detected in the ancient genome of 45’000 years old *Homo sapiens* from Ust’-Ishim, and were found to diverge between Neanderthals and Denisova (Table 1 and Supplementary Table S8). Interestingly, ancient Neanderthal genomes apparently carry the same allele (A) as *Pan* at rs4987076 whereas the alternative allele (G) was found for Denisova, and the opposite pattern is observed at rs4986990 (both *Pan* and Denisova carry G, while ancient Neanderthal genomes carry A). Thus, both the 445 A/G (rs4987076) and the 459 G/A (rs4986990) polymorphisms could potentially either pre-date the divergence among hominids or represent a case of independent parallel mutation(s) in hominins and in other great ape lineages. In turn, at position 640 G/T (rs4986783), all great apes and Neanderthals were found to carry G, whereas the alternative allele (T) was only observed in Denisova. In humans today, the derived alleles (respectively A, A and G) at these three SNPs (respectively, rs4987076, rs4986990 and rs4986783) characterize human haplotypes NAT1*11A and NAT1*11B. A previous study estimated that the coalescence of NAT1*11A with other major human *NAT1* haplotypes (NAT1*3, NAT1*4, and NAT1*10) dates back to 2 million years ago, leading the authors to suggest that some *NAT1* diversity in the genome of modern humans may have persisted from a structured ancestral population (Patin *et al.* 2006a). Since SNPs 445 A/G and 459 G/A are shared between humans and orangutans (SNP 445 A/G being also shared with gorillas), their coalescence times could be even older and likely pre-date the divergence among hominids. When considered together, the three SNPs define three major combinations in hominids, GGT, AGG, and AAG. The most frequent combination in humans, GGT, which characterizes most human haplotypes (except those of the NAT1*11 series and NAT1*30), was not observed in the other great apes. Instead, all haplotypes in chimpanzees and bonobos carry the AGG combination, which has an estimated frequency of 70% in gorillas, and at least 55% in orangutans, but has not been reported so far in human populations. Such observations could suggest that the AGG haplotype was present in the ancestors of hominids (the reference sequences of rhesus (*Macaca mulatta*) and cynomolgus (*M. fascicularis*) macaques, rheMac8 and macFas5, also carry the AGG combination), and was lost at some point in the human lineage, possibly after the divergence from Denisova and Neanderthal. Indeed, Denisova’s ancient genome is defined as GGT, while the Altai and Vindija Neanderthal genomes are reported with the AAG combination, and the genome of 45’000 years old *Homo sapiens* from Ust’-Ishim is heterozygous at the three positions (Supplementary Table S8). Nowadays, the human reference haplotype NAT1*4, together with other haplotypes carrying GGT at the three SNPs (*e.g.*, human NAT1*3 and NAT1*10), have an average cumulated frequency of 95% in all human populations studied so far, whereas haplotypes with AAG (the human NAT1*11 series) are observed at very low frequencies in populations from Africa, Asia, Europe and New Guinea (Patin *et al.* 2006a; Mortensen *et al.* 2011).

*NAT2* stands in sharp contrast with *NAT1*, mainly because of the high levels of *NAT2* polymorphism in humans (Supplementary Table S4, 55 polymorphic positions recorded for humans at *NAT2*, which represents twice as many compared to *NAT1*). The proportion of *NAT2* non-synonymous SNPs in humans (75%) seems higher than that found in *Pan* (56%), orangutans (58%), and gorillas (38%).

It is noticeable that among the four *NAT2* polymorphisms shared between hominid species (or five if considering modern humans and Neanderthals as distinct species), all are shared with humans. Two of these are non-synonymous polymorphisms in humans shared with the *Pan* genus: G/A at human cds position 191 (rs1801279) observed in bonobos, and C/T at 578 (rs79050330) observed in Western chimpanzees (Table 1). Humans were also found to share two SNPs with gorillas: synonymous C/T at 345 (rs45532639) and non-synonymous A/G at 506 (rs200585149). These shared SNPs are rare polymorphisms in humans (Sabbagh *et al.* 2008; The Genomes Project Consortium 2012), with the exception of 191 G/A (rs1801279), the signature mutation of human haplotypes of the NAT2*14 series (NAT2*14A/B/E/H/L), observed mostly among African populations (Patin *et al.* 2006a; Mortensen *et al.* 2011; Podgorná *et al.* 2015). None of the polymorphisms detected in orangutans are shared with other great apes. Remarkably, the common human synonymous C/T SNP at 282 (rs1041983) was found polymorphic in one Neanderthal ancient genome (Altai, Supplementary Table S8). This SNP is, up to now, the sole *NAT* exonic position recorded as polymorphic in a non-anatomically modern human ancient genome. Note that due to an apparent inconsistency between the ancient genome browser at the Max Planck Institute for Evolutionary Anthropology and the UCSC genome browser, we could not conclude whether the *NAT2* SNP 803 A/G (rs1208) differentiates the Denisova genome from Neanderthals and all other hominids (Supplementary Table S8). This mutation is common in most modern human populations except in East Asia, and despite being non-synonymous it does not alter the enzyme’s activity (Zang *et al.* 2007).

Sequence diversity at the *NATP* pseudogene in hominids differs from that of its functional paralogs by the presence of several insertions and deletions (InDels) that could be evidenced in the Sanger sequenced samples of this study, and most of which mark divergence between species (Supplementary Table S5). In addition to InDels, high levels of single nucleotide polymorphisms also characterize this pseudogene in hominids (45 SNPs detected in humans, 13 in gorillas, 19 in orangutans, and 17 at least in the genus *Pan*), but similarly to the functional *NAT1* and *NAT2* genes, little sharing between species was found (Table 1). Ancient *Homo* genomes also reflect the high level of hominid polymorphism at *NATP* (Supplementary Table S8). Besides three modern human SNPs that were also found heterozygous in the ancient Ust’-Ishim *Homo sapiens* sample, three positions at least apparently differ between Neanderthals and Denisova, a proportion similar to that of *NAT1*. We also note that an A/G polymorphism is reported in Denisova (at position 18’228’182) which, according to our dataset, has not been detected in any other hominid species.

Within the *Pan* genus, the largest group of genotyped samples in this study (and in particular the Western chimpanzee sub-species, *Pan troglodytes verus*), 40 segregating sites were observed in total for the three *NAT* genes. Among these 40 sites, only 15 were found in Western chimpanzees (Table 2; see also Supplementary File S1, and Supplementary Tables S3, S4 and S5). We detected 32 polymorphic positions in common chimpanzees (*P. troglodytes*), but only five are shared by all *Pan troglodytes* sub-species (Table 2).

None of the polymorphic positions shared between all common chimpanzees is observed in bonobos (*P. paniscus*). In this latter species, only five polymorphic sites were identified in the three *NAT* genes, one of which (at 18’079’925 in *NAT1*) is shared with Western chimpanzees and the other (at 18’228’659 in *NATP*) with Central and Eastern chimpanzees. Six positions were apparently fixed on the derived allele in bonobos, one of them (at 18’228’285 in *NATP*) being polymorphic in common chimpanzees.

### NAT haplotypes in Pan

The *Arylamine N-acetyltransferase* gene nomenclature committee (Hein *et al.* 2008) currently lists 28 human *NAT1* haplotypes encoding a dozen variant NAT1 proteins, some of which are associated with lower enzymatic activity than the reference human NAT1*4 haplotype, while the number of documented human *NAT2* haplotypes listed is 108 and the number of encoded variant NAT2 proteins comes close to 70, about 15 of which are involved in a “slow” acetylation phenotype (http://nat.mbg.duth.gr). In humans, as reviewed in (Sabbagh *et al.* 2018), frequency distributions of haplotypes encoding NAT2 proteins are highly variable among populations (see for instance Figure 2 in (Sabbagh *et al.* 2018)), whereas for *NAT1*, frequency distributions in most human populations are dominated by only two major haplotypes (with cumulated frequencies varying between 85% and 100%), that differ by two SNPs in the 3′UTR region and whose relative phenotypic effect is considered as moderate (haplotype NAT1*10 probably enhancing protein expression relative to the most common haplotype, NAT1*4, (Hein *et al.* 2018)).

Knowledge of *NAT* diversity within other great ape species is at present lacking. We thus inferred haplotypes at the three homologous *NAT* genes for individuals belonging to the genus *Pan* (due to the small size of available samples, such inference was not possible for gorillas and orangutans), so as to characterize *Pan NAT* diversity, and predict the functional consequences of non-synonymous mutations. The PHASE analysis of the 108 *Pan* genotypes (94 chimpanzees and 14 bonobos) led to the inference of 12 *Pan* haplotypes for *NAT1*, 10 for *NAT2*, and 19 for *NATP* (Table 3).

**Table 3:** Haplotypes of the three *NAT* gene paralogs in the genus *Pan*

*NAT1*										
Position*^a^*	79’632	79’703	79’859	79’897	79’925	80’014	80’074	80’153	80’316	80’345
SNP*^b^*	G76A	C147T	C303T	T341C	T369C	C458T	A518C	T597G	G760C	A789G
Amino acid change	D26N			I114T			E173A	I199M	E254Q	I263M
Haplotypes										
NAT1*1	G	C	C	T	T	C	A	T	G	A
NAT1*2	.	T	.	.	.	.	.	.	.	.
NAT1*3	.	.	.	.	.	.	.	.	.	G
NAT1*4	.	T	.	.	.	.	.	G	.	.
NAT1*5	A	.	.	.	.	.	.	.	.	.
NAT1*6	.	.	.	.	C	.	.	.	.	.
NAT1*7	.	.	.	.	.	.	.	.	C	.
NAT1*8	.	.	.	C	.	.	.	.	.	.
NAT1*9	.	.	.	.	.	T	C	.	.	.
NAT1*10	A	T	.	.	.	.	.	.	.	.
NAT1*11	.	.	.	.	.	.	C	.	.	.
NAT1*12	.	.	T	.	.	.	.	.	.	.

*NAT2*									
Position*^c^*	57’549	57’585	57’658	57’704	58’027	58’091	58’302	58’447	58’462
SNP*^b^*	T36C	A72C	G145A	G191A	A514G	C578T	T789G	A934G	C949G	
Amino acid change		L24F	E49K	R64Q	N172D	T193M			
Haplotypes									
NAT2*1	T	A	G	G	A	C	T	A	C
NAT2*2	.	.	.	.	.	T	.	.	.
NAT2*3	.	.	.	.	.	.	G	.	.
NAT2*4	.	.	.	.	.	.	.	G	.
NAT2*5	.	.	.	.	.	.	.	.	G
NAT2*6	.	.	.	.	G	.	.	.	.
NAT2*7	.	.	A	.	.	.	.	G	.
NAT2*8	.	.	A	A	.	.	.	G	.
NAT2*9	.	C	A	.	.	.	.	G	.
NAT2*10	C	.	.	.	G	.	.	.	.

*NATP*										
Position*^c^*	28’146	28’189	28’238	28’242	28’279	28’285	28’304	28’368	28’404	28’501
SNP*^b^*	31	74	123	127	164	170	189	253	289	386
Haplotypes										
NATP*1	G	C	A	A	T	T	C	C	C	T
NATP*2	.	.	.	.	.	.	.	T	.	.
NATP*3	.	.	.	.	.	.	.	T	.	.
NATP*4	.	.	.	.	.	.	.	T	.	.
NATP*5	.	.	.	.	.	.	.	T	.	.
NATP*6	.	.	.	.	.	.	.	T	.	C
NATP*7	.	.	G	.	.	.	.	T	.	.
NATP*8	.	.	.	.	.	.	.	T	.	C
NATP*9	.	.	.	.	.	.	.	T	.	C
NATP*10	.	.	.	.	.	.	.	T	.	C
NATP*11	.	.	.	.	.	A	.	T	.	C
NATP*12	.	.	.	.	.	A	.	T	.	C
NATP*13	.	.	.	.	.	A	.	T	T	C
NATP*14	.	.	.	.	.	A	.	T	T	C
NATP*15	.	.	.	.	.	A	.	T	T	C
NATP*16	.	T	.	.	.	A	.	T	.	C
NATP*17	T	.	.	.	.	A	.	T	.	C
NATP*18	.	.	.	G	C	A	T	T	.	C
NATP*19	.	.	.	G	C	A	T	T	.	C

*NATP* (continued)										
Position*^c^*	28’543	28’560	28’575	28’576	28’582	28’614	28’659	28’660	28’661	28’748
SNP*^b^*	428	445	460	461	467	499	544	545	546	633
Haplotypes										
NATP*1	A	C	A	G	G	T	G	C	G	T
NATP*2	.	.	.	.	.	.	.	.	.	.
NATP*3	.	.	.	.	.	.	.	.	A	.
NATP*4	.	.	.	.	T	.	.	.	.	.
NATP*5	.	A	.	.	.	.	.	.	.	.
NATP*6	.	.	.	.	.	.	.	.	.	.
NATP*7	.	.	.	.	.	.	.	.	.	.
NATP*8	.	.	.	.	.	G	.	.	.	.
NATP*9	.	.	.	.	.	G	.	.	.	.
NATP*10	.	.	.	.	.	G	A	.	.	.
NATP*11	.	.	.	.	.	.	.	.	.	.
NATP*12	.	.	.	.	.	.	.	.	.	.
NATP*13	.	.	.	.	.	G	.	.	.	C
NATP*14	G	.	.	.	.	.	.	.	.	.
NATP*15	G	.	.	.	.	G	.	.	.	.
NATP*16	.	.	.	.	.	G	.	.	.	.
NATP*17	.	.	.	.	.	.	.	.	.	.
NATP*18	.	.	G	A	.	G	.	T	.	.
NATP*19	.	.	G	A	.	G	A	T	.	

*NATP* (continued)				
Position*^c^*	28’771	28’959	29’057	29’103
SNP*^b^*	656	844	942	988
Haplotypes										
NATP*1	C	T	G	A
NATP*2	.	.	.	.
NATP*3	.	.	.	.
NATP*4	.	.	.	.
NATP*5	.	.	.	.
NATP*6	.	.	.	.
NATP*7	.	.	.	.
NATP*8	.	.	.	.
NATP*9	T	.	.	.
NATP*10	T	.	.	.
NATP*11	.	.	.	.
NATP*12	.	C	.	.
NATP*13	T	.	.	.
NATP*14	T	.	.	.
NATP*15	T	.	.	.
NATP*16	T	.	.	T
NATP*17	.	.	.	.
NATP*18	.	.	A	.
NATP*19	.	.	A	.

a Position (+18’000’000) on GRCh37/hg19.

b SNP position relative to the coding exon of *NAT1*, *NAT2*, or its paralog sequence on *NATP* (starts at position 1).

c Position (+18’200’000) on GRCh37/hg19.

**Figure 1:**
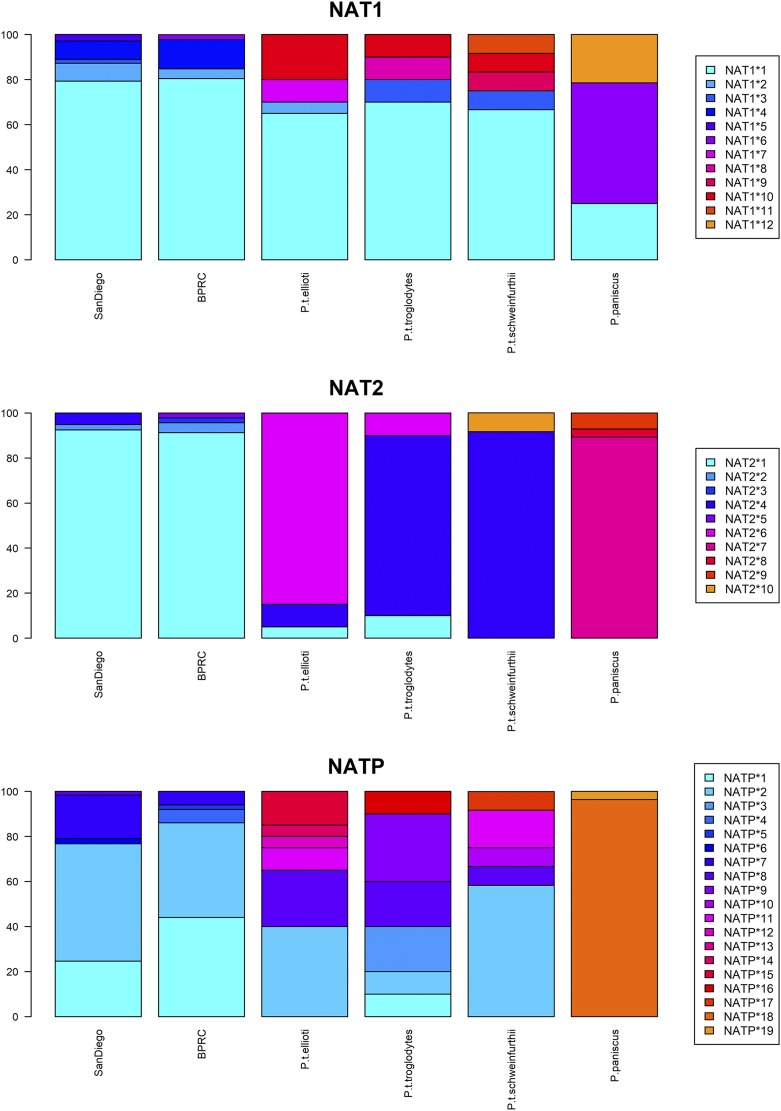
Haplotype frequency distributions at the three *NAT* genes in *Pan* species and sub-species.

We found dissimilar frequency distributions between the three *NAT* genes in all *Pan* species (Table 4 and Figure 1; haplotype counts in the total samples including related individuals are provided in Supplementary Table S9). As reported in Table 4, none of these frequency distributions were found to deviate from Hardy-Weinberg equilibrium, after correction for multiple testing.

**Table 4:** *NAT* haplotype frequencies (%) estimated in the different species and sub-species of the genus *Pan* and results of Hardy-Weinberg equilibrium tests.

*Pan* species and sub-species						
	*P. t. verus* (Western chimpanzee)					
	San Diego sample*^a^*	BPRC sample	*P. t. ellioti* (Nigeria-Cameroon chimpanzee)	*P. t. troglodytes* (Central chimpanzee)	*P. t. schweinfurthii* (Eastern chimpanzee)	*P. paniscus* (Bonobo)
***Pan NAT1* haplotypes**						
NAT1*1	79.33 (1.71)	80.43	65.00	70.00	66.70	25.00
NAT1*2	7.97 (0.94)	4.35	5.00	0	0	0
NAT1*3	1.59 (1.38)	0	0	10.00	8.33	0
NAT1*4	8.33 (0)	13.04	0	0	0	0
NAT1*5	2.78 (0)	0	0	0	0	0
NAT1*6	0	2.17	0	0	0	53.60
NAT1*7	0	0	10.00	0	0	0
NAT1*8	0	0	0	10.00	0	0
NAT1*9	0	0	0	0	8.33	0
NAT1*10	0	0	20.00	10.00	8.33	0
NAT1*11	0	0	0	0	8.33	0
NAT1*12	0	0	0	0	0	21.40
Total (2n chromosomes)	36	46	20	10	12	28
Hardy-Weinberg test*^b^*:						
*H*o	0.36 (0.03)	0.26	0.50	0.60	0.50	0.71
*H*e	0.37 (0.03)	0.34	0.55	0.53	0.58	0.63
*P*-value	∈ [0.39 ; 0.64]	0.20	0.35	> 0.99	0.51	0.45
***Pan NAT2* haplotypes**						
NAT2*1	92.49 (1.27)	91.30	5.00	10.00	0	0
NAT2*2	2.41 (0.94)	4.35	0	0	0	0
NAT2*3	0	2.17	0	0	0	0
NAT2*4	5.10 (1.03)	0	10.00	80.00	91.70	0
NAT2*5	0	2.17	0	0	0	0
NAT2*6	0	0	85.00	10.00	0	0
NAT2*7	0	0	0	0	0	89.3
NAT2*8	0	0	0	0	0	3.57
NAT2*9	0	0	0	0	0	7.14
NAT2*10	0	0	0	0	8.33	0
Total (2n chromosomes)	36	46	20	10	12	28
Hardy-Weinberg test*^b^*:						
*H*o	0.15 (0.03)	0.17	0.30	0.20	0.17	0.21
*H*e	0.15 (0.02)	0.17	0.28	0.38	0.17	0.20
*P*-value	∈ [0.08 ; > 0.99 ]	> 0.99	> 0.99	0.11	> 0.99	> 0.99
***Pan NATP* haplotypes**						
NATP*1	24.61 (2.46)	44.00	0	10.00	0	0
NATP*2	52.14 (1.7)	42.00	40.00	10.00	58.30	0
NATP*3	0	0	0	20.00	0	0
NATP*4	0	6.00	0	0	0	0
NATP*5	0	2.00	0	0	0	0
NATP*6	2.32 (1.03)	0	0	0	0	0
NATP*7	19.33 (2.21)	6.00	0	0	0	0
NATP*8	1.59 (1.38)	0	25.00	20.00	8.33	0
NATP*9	0	0	0	30.00	0	0
NATP*10	0	0	0	0	8.33	0
NATP*11	0	0	10.00	0	16.67	0
NATP*12	0	0	5.00	0	0	0
NATP*13 *^c^*	0	0	0	0	0	0
NATP*14	0	0	5.00	0	0	0
NATP*15	0	0	15.00	0	0	0
NATP*16	0	0	0	10.00	0	0
NATP*17	0	0	0	0	8.33	0
NATP*18	0	0	0	0	0	96.43
NATP*19	0	0	0	0	0	3.57
Total (2n chromosomes)	36	50	20	10	12	28
Hardy-Weinberg test*^b^*:						
*H*o	0.64 (0.04)	0.60	0.90	0.60	0.67	0.07
*H*e	0.65 (0.01)	0.64	0.78	0.89	0.67	0.07
*P*-value	∈ [0.09 ; > 0.99 ]	0.06	**0.03** *^d^*	0.15	0.76	> 0.99

a Average over the 122 sub-samples (see text), standard deviation in brackets.

b Test for departure from Hardy-Weinberg equilibrium; *H*o: observed heterozygosity, *H*e: expected heterozygosity (equivalent to gene diversity). The only significant deviation from equilibrium (heterozygote excess at *NATP* in *P. t. ellioti*) is shown in bold.

c Haplotype NATP*13, which combines SNPs at 170, 253, 289, 386, 499, 633, and 656 (Table 3), was inferred only for the genotype of the hybrid *P. t. verus/troglodytes* individual.

d *P*-value > 0.05 after correction for multiple testing.

At *NAT1*, haplotype frequency distributions in all chimpanzee sub-species are characterized by a single major haplotype (NAT1*1 occurring at frequencies between 65% and 80%) along with three to four other haplotypes at lower frequencies (Table 4 and Figure 1). By contrast, NAT1*6 is the more frequent haplotype in bonobos (54%), followed by NAT1*1 and NAT1*12 (25% and 21%, respectively). When measured by Φ_ST_ fixation indices, which take into consideration the molecular diversity of haplotypes, significant differentiation was only found between bonobos and the other *Pan* (Supplementary Table S6). On the other hand, it is noteworthy that in bonobos, the two haplotypes NAT1*6 and NAT1*12 observed besides NAT1*1 differ from it only by a single synonymous mutation (Table 3 and Supplementary Figure S2). In other words, 100% of proteins expressed by *NAT1* in bonobos are identical to the product of the NAT1*1 haplotype predominant in all chimpanzee sub-species. In turn, most of the other *NAT1* haplotypes detected in these sub-species differ from NAT1*1 by one non-synonymous change, resulting in the existence of seven distinct NAT1 proteins: at least four in each of the Western (*P. t. verus*), Central (*P. t. troglodytes*), and Eastern (*P. t. schweinfurthii*) sub-species, and three in the Nigeria-Cameroon (*P. t. ellioti*) chimpanzees, according to our observations (Supplementary Table S10).

At *NAT2*, a single frequent haplotype is observed in all *Pan* species, along with one to three less frequent haplotypes (Table 4 and Figure 1). In contrast to *NAT1*, the frequency of the most prevalent *NAT2* haplotype is higher (80–92.5%), and it differs between species and sub-species: it is NAT2*1 in the Western (*P. t. verus*), NAT2*4 in the Central (*P. t. troglodytes*) and Eastern (*P. t. schweinfurthii*), and NAT2*6 in the Nigeria-Cameroon (*P. t. ellioti*) chimpanzee sub-species, and NAT2*7 in the bonobos (*P. paniscus*). Also in contrast to *NAT1*, no *NAT2* haplotype was found shared between chimpanzees and bonobos. Thus, unsurprisingly, significant differentiation was found not only between bonobos and the other *Pan*, but also between all chimpanzee sub-species, except between Central (*P. t. troglodytes*) and Eastern (*P. t. schweinfurthii*) chimpanzees (Supplementary Table S6). On the other hand, although haplotype NAT2*4 is the predominant haplotype in Central and Eastern chimpanzees, it only differs by one synonymous mutation from the most prevalent NAT2*1 haplotype in Western (*P. t. verus*) chimpanzees (Table 3 and Supplementary Figure S3). This suggests little differentiation between these three Western, Central, and Eastern sub-species at the level of *NAT2* gene products. Indeed, the 10 *NAT2* haplotypes detected in *Pan* translate into six distinct NAT2 proteins, of which we observed two in each of the chimpanzee subspecies, and three in bonobos (Supplementary Table S10). In fact, both the commonest haplotype in Nigeria-Cameroon chimpanzees (NAT2*6 in *P. t. ellioti*) and all haplotypes in bonobos differ from most other *Pan NAT2* haplotypes by at least one non-synonymous change.

Finally, the *NATP* pseudogene haplotypes are more evenly distributed than those of the two functional genes in all the chimpanzee sub-species, with two or more frequent *NATP* haplotypes (Table 4 and Figure 1). By contrast, in bonobos, only two haplotypes were found, one of which with a frequency of 96% (NATP*18), and none is shared with chimpanzees. However, substantial haplotype sharing is observed between chimpanzee sub-species, notably for haplotypes NATP*2 and NATP*8. Analyses of molecular variance detected a significant level of genetic differentiation only between bonobos and all chimpanzees, and among the latter, between Western chimpanzees (*P. t. verus*) and the other sub-species (Supplementary Table S6). A high level of sequence diversity at *NATP* may explain these complex results. Indeed, the median-joining network of *NATP* haplotypes (Supplementary Figure S4) displays two reticulations and a few rather divergent haplotypes (*e.g.*, NATP*14, NATP*15, and NATP*16), which raises the possibility that some *Pan* haplotypes were unsampled.

### Predicted functional differences among NAT1 and NAT2 haplotypes in Pan

We chose Pan NAT1*1 and Pan NAT2*4, the basal haplotypes in the median-joining networks of *NAT1* and *NAT2* haplotypes, respectively (Supplementary Figures S2 and S3), as reference sequences to predict the functional impact of *NAT* mutations in *Pan* using PolyPhen, SIFT and PANTHER cSNP Scoring, three online software tools.

Four of the 11 *Pan NAT1* haplotypes derived from NAT1*1 were predicted to be damaging with more or less confidence (Table 5): NAT1*4 and NAT1*7, each predicted by two tools, and NAT1*3 and NAT1*8 by one tool only. The other seven haplotypes were either predicted as not damaging or only differ from the others by synonymous mutations. The cumulated frequencies of potentially damaging haplotypes could thus reach the values of 10–13% in Western (*P. t. verus*) chimpanzees, 8–10% in Eastern (*P. t. schweinfurthii*) and Nigeria-Cameroon (*P. t. ellioti*) chimpanzees, and up to 20% in Central (*P. t. troglodytes*) chimpanzees, at best (Table 4, Figure 1, and Supplementary Figure S2). If confirmed, these results suggest that a sizeable proportion of chimpanzees may have a moderately reduced NAT1 acetylation capacity. Nevertheless, the frequency profiles of *NAT1* in the *Pan* species and sub-species are characterized by a majority of haplotypes that are not damaging, and thus translating into a similar enzymatic activity.

**Table 5:** Predictions of the effect of mutations between *Pan NAT1* and *NAT2* coding sequences according to PolyPhen, SIFT and PANTHER cSNP Scoring.

			PolyPhen		SIFT		PANTHER cSNP Scoring	
Haplotypes	cDNA	protein	Score*^a^*	Prediction*^b^*	Score*^c^*	Prediction*^d^*	PSEP*^e^*	Prediction*^f^*
*Pan NAT1* (reference NAT1*1)*^g^*								
NAT1*3	A789G	I263M	0.279 (0.91-0.88)	B	0.08 (3.08, 80)	T	220	POD
NAT1*4	T597G	I199M	0.369 (0.9-0.89)	B	0.01 (3.07, 81)	A	220	POD
NAT1*5	G76A	D26N	0.377 (0.9-0.89)	B	0.1 (3.08, 76)	T	91	B
NAT1*7	G760C	E254Q	0.892 (0.82-0.94)	POD	0.07 (3.08, 80)	T	455	PRD
NAT1*8	T341C	I114T	0.099 (0.93-0.85)	B	0.06 (3.07, 81)	T	220	POD
NAT1*11	A518C	E173A	0.013 (0.96-0.78)	B	0.17 (3.07, 81)	T	30	B
*Pan NAT2* (reference NAT2*4)*^h^*								
NAT2*2	C578T	T193M	1 (0.00-1.00)	PRD	0 (3.07, 51)	A	456	PRD
NAT2*6	A514G	N172D	0.001 (0.99-0.15)	B	0.26 (3.07, 51)	T	220	POD
NAT2*7	G145A	E49K	0.002 (0.99-0.3)	B	0.5 (3.07, 50)	T	324	POD
NAT2*8*^i^*	G191A	R64Q	1.00 (0.00-1)	PRD	0 (3.07, 50)	A	4200	PRD
NAT2*9*^i^*	A72C	L24F	1 (0.00-1)	PRD	0 (3.07, 50)	A	4200	PRD

a PolyPhen score: probability that a substitution is damaging; sensibility and specificity in brackets.

b PolyPhen prediction: “benign” (B), “possibly damaging” (POD), “probably damaging” (PRD).

c SIFT score: probability that a substitution is tolerated; median sequence information and number of sequences used for the prediction in brackets.

d SIFT prediction: T: “tolerated” (T), A: “affect protein function” (A).

e PANTHER cSNP Scoring PSEP (position-specific evolutionary preservation): length of time (in millions of years) of preservation of a position.

f PANTHER cSNP Scoring prediction: “probably damaging” (PRD), “possibly damaging” (POD), “probably benign” (B).

g The reference *Pan NAT1* haplotype used is the basal haplotype in the network of *NAT1* sequences (Supplementary Figure S2).

h The reference *Pan NAT2* haplotype used is the basal haplotype in the network of *NAT2* sequences (Supplementary Figure S3). Since NAT2*1 differs from NAT2*4 at a single position located 61 bp downstream the coding exon relative to the stop codon (A934G, Table 3), the two haplotypes likely translate into a similar gene product, so that haplotypes deriving from NAT2*1 could be predicted using NAT2*4 as a reference. Instead, both haplotypes NAT2*8 and NAT2*9 derive from NAT2*7, which differs from the basal haplotype at SNP G145A (E49K, Table 3). Thus, for the non-synonymous mutations defining NAT2*8 and NAT2*9, predictions were performed using NAT2*7 as a reference.

i Haplotypes NAT2*8 and NAT2*9 both bear the G145A mutation defining haplotype NAT2*7. Since the prediction tools do not allow the simultaneous specification of two substitutions, we ran the prediction tools for G191A and A72C against NAT2*7 as a reference, instead of NAT2*4.

As displayed in the *Pan NAT2* network (Supplementary Figure S3), nine haplotypes derive from the NAT2*4 basal haplotype, three of which reaching high frequencies, *i.e.*, NAT2*1, NAT2*6, and NAT2*7 (Table 4, Figure 1 and Supplementary Figure S3). Three low frequency haplotypes out of the nine stemming from the basal haplotype were predicted as damaging with high confidence by the three tools: NAT2*2, only observed in *P. t. verus*, and NAT2*8 and NAT2*9, the two haplotypes observed only in *P. paniscus*. Interestingly, the mutations that define NAT2*2 and NAT2*8 both occur at positions that are also polymorphic in humans (positions 578, rs79050330 and 191, rs1801279, respectively). The effect of SNP 191 G/A on *NAT2* enzymatic activity in humans is known; it defines the human NAT2*14 haplotype series that is associated with a slow acetylation phenotype (see Supplementary File S1), which is thus consistent with our prediction results. That of SNP 578 T/C (which is associated in humans with haplotypes NAT2*5P, NAT2*12E, and NAT2*13B) is unknown. The prediction tools do not return clear-cut results for the mutations defining NAT2*6 (derived from NAT2*1, and predominant in *P. t. ellioti*) and NAT2*7 (derived from NAT2*4, and predominant in *P. paniscus*) (namely A/G at position 514 and G/A at 145, respectively), predicted as possibly damaging by PANTHER cSNP Scoring only, and with a low confidence. This suggests that their potential effects are either less damaging than those of mutations defining NAT2*2, NAT2*8, and NAT2*9 (*i.e.*, rs79050330 C/T at 578, rs1801279 G/A at 191, and SNP A/C at 72), or that they depend on the substrate, as is known for some human polymorphisms (Hein *et al.* 2006) (see Supplementary File S1). In contrast to Western (*P. t. verus*) chimpanzees, where the cumulated frequencies of NAT2*2, NAT2*8 and NAT2*9 are lower than 5%, they reach 10% in bonobos (Table 4 and Figure 1). If confirmed, our prediction results could thus indicate that a significant proportion of bonobos (but a smaller proportion of Western chimpanzees) could have a slow NAT2 acetylation phenotype.

### NAT genetic diversity and tests of selective neutrality in Pan

**Figure 2:**
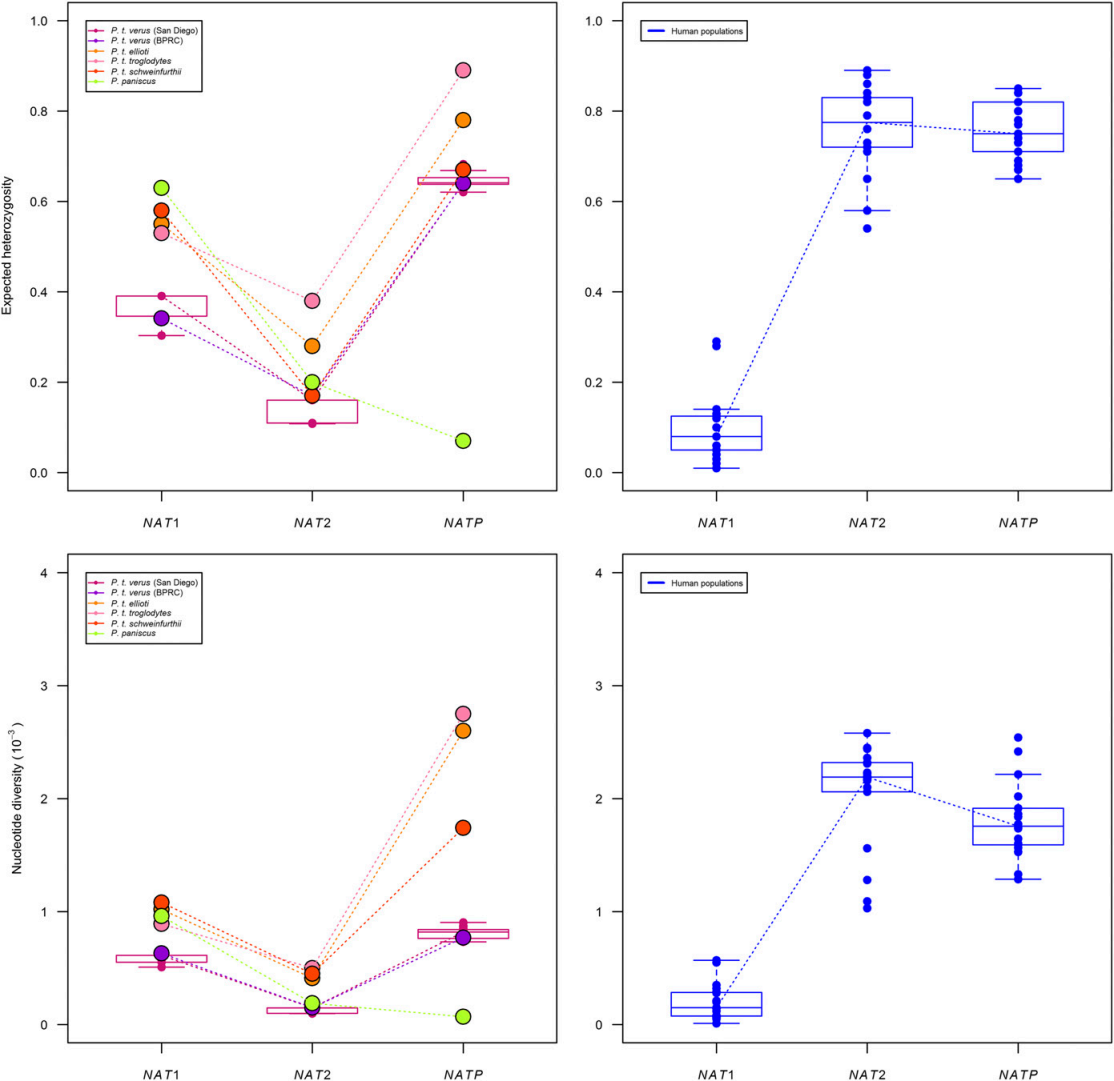
Expected heterozygosity (*h*) and nucleotide diversity (*π* x 10^−3^) at the three *NAT* genes in *Pan* species and sub-species (left panes) and in human populations (right panes). The variation of values among the 122 San Diego *P. t. verus* sub-samples (left panes) and among human populations samples (right panes) are shown by boxplots. The dotted lines were added to the graphs to highlight inter-locus variation. For *Pan*, *P*-values of Wilcoxon rank-sum tests after adjustment for multiple testing (and using only the average value for the San Diego sample) were of 0.039 for *NAT1* *vs.* *NAT2*, 0.065 for *NAT1* *vs.* *NATP*, and 0.065 for *NAT2* *vs.* *NATP*, respectively, for differences in expected heterozygosity (*h*), and of 0.0065 for *NAT1* *vs.* *NAT2*, 0.5887 for *NAT1* *vs.* *NATP*, and 0.0974 for *NAT2* *vs.* *NATP*, respectively, for differences in nucleotide diversity (*π*). When restricting the tests to the chimpanzee (*P. troglodytes*) data only, *P*-values were of 0.012 for *NAT1* *vs.* *NAT2*, 0.222 for *NAT1* *vs.* *NATP*, and 0.012 for *NAT2* *vs.* *NATP*, respectively, for expected heterozygosity, and of 0.036 for *NAT1* *vs.* *NAT2*, 0.018 for *NAT1* *vs.* *NATP*, and 0.018 for *NAT2* *vs.* *NATP*, respectively for nucleotide diversity. For human populations, adjusted Wilcoxon rank-sum tests *P*-values for differences in both expected heterozygosity and nucleotide diversity were all < 0.0001 in the comparisons of *NAT1* *vs.* *NAT2*, and *NAT1* *vs.* *NATP*, and of 0.58 and 0.048 for expected heterozygosity and nucleotide diversity, respectively, in the *NAT2* *vs.* *NATP* comparison.

We found that the Western (*P. t. verus*) chimpanzee sub-species has the lowest diversity among the *Pan* genus for *NAT1* (Table 6 and Figure 2). Indeed, expected heterozygosity (*h*) ranges from 0.34 in Western chimpanzees (BPRC sample) to 0.63 in bonobos (*P. paniscus*), and nucleotide diversity (reported as *π* x 10^−3^) from 0.58 in Western chimpanzees to 1.08 in Eastern (*P. t. schweinfurthii*) chimpanzees. A significant deviation from selective neutrality and demographic equilibrium due to homozygosity excess was found for the Central (*P. t. troglodytes*) and Eastern (*P. t. schweinfurthii*) chimpanzee sub-species with one test of selective neutrality, *i.e.*, the Ewens-Watterson test. However, although highly significant, these results must be considered with caution as they may represent artifacts due to the low sample sizes for these two sub-species (only five and six individuals, respectively, Table 6). No other neutrality or demographic equilibrium test returned any significant result.

**Table 6:** Genetic diversity and results of selective neutrality tests for the three *NAT* gene paralogs in the different species and sub-species of the genus *Pan*, with equivalent estimates in human populations shown in the last column.

	*Pan* species and sub-species						
	*P. t. verus* (Western chimpanzee)						*Homo sapiens*
	San Diego sample*^a^*	BPRC sample	*P. t. ellioti* (Nigeria-Cameroon)	*P. t. troglodytes* (Central)	*P. t. schweinfurthii* (Eastern)	*P. paniscus* (Bonobo)	(human populations average)*^b^*
***Pan NAT1***							
Total (2N chromosomes)	36	46	20	10	12	28	119.7
Number of usable positions	903	903	898	898	898	898	903
Number of segregating sites (*S*)	3.57 (0.5)	3	3	4	5	2	3.75
Number of haplotypes (*k*)	4.57 (0.5)	4	4	4	5	3	3.5
Expected heterozygosity (*h*)	0.37 (0.03)	0.34	0.55	0.53	0.58	0.63	0.095
Nucleotide diversity (*π*) x 10^−3^	0.58 (0.03)	0.63	1.02	0.89	1.08	0.96	0.187
Ewens-Watterson test*^c^*:							
*F*o	0.64 (0.026)	0.67	0.48	0.52	0.47	0.40	0.902
*F*e	0.45 (0.041)	0.52	0.44	0.37	0.30	0.59	0.618
*P*-value	∈ [0.84 ; 0.95]	0.82 (0.18)	0.70 (0.10)	**> 0.99 (>0.99)**	**> 0.99 (>0.99)**	0.09 (0.25)	9 (**8**)/19
Tajima’s *D* test*^d^*:							
*D*	−0.91 (0.17)	−0.35	0.24	−1.67	−1.53	1.43	−1.475
*P*-value	∈ [0.112 ; 0.282]	0.398 (0.642)	0.642 (0.099)	0.031 (0.061)	0.056 (0.056)	0.917 (0.752)	11 (**1**) / 18
Fu’s *F*_S_ test*^e^*:							
*F*_S_	−1.64 (0.47)	−0.59	−0.20	−1.35	−1.98	1.19	−2.849
*P*-value	∈ [0.038 ; 0.197]	0.321 (0.642)	0.399 (0.901)	0.043 (0.061)	0.024 (0.048)	0.742 (0.742)	8 (**3**) / 18
***Pan NAT2***							
Total (2N chromosomes)	36	46	20	10	12	28	137.8
Number of usable positions	1’115	1’115	1’091	1’091	1’091	1’112	1'115
Number of segregating sites (*S*)	1.87 (0.34)	3	2	2	3	2	9.78
Number of haplotypes (*k*)	2.87 (0.34)	4	3	3	2	3	10.7
Expected heterozygosity (*h*)	0.15 (0.02)	0.17	0.28	0.38	0.17	0.20	0.761
Nucleotide diversity (*π*) x 10^−3^	0.13 (0.02)	0.15	0.41	0.50	0.45	0.19	2.041
Ewens-Watterson test*^c^*:							
*F*o	0.86 (0.02)	0.84	0.74	0.66	0.85	0.80	0.249
*F*e	0.63 (0.05)	0.52	0.56	0.49	0.70	0.59	0.291
*P*-value	∈ [0.75 ; **> 0.99**]*^f^*	**0.98** (0.96)	0.90 (0.69)	**> 0.99 (>0.99)**	**> 0.99 (>0.99)**	0.92 (0.92)	0 / 18
Tajima’s *D* test*^d^*:							
*D*	−1.26 (0.19)	−1.58	−0.44	−0.69	−1.63	−1.24	0.639
*P*-value	∈ [0.034 ; 0.158]	**0.020** (0.040)	0.337 (0.521)	0.237 (0.246)	**0.019** (0.039)	0.076 (0.076)	2 (0) / 18
Fu’s *F*_S_ test*^e^*:							
*F*_S_	−1.84 (0.55)	−3.43	−0.377	−0.59	1.054	−1.59	−0.765
*P*-value	∈ [**0.004** ; 0.129]*^g^*	**0.001 (0.003)**	0.260 (0.521)	0.123 (0.246)	0.595 (0.595)	**0.015 (0.015)**	2 (0) / 18
***Pan NATP***							
Total (2N chromosomes)	36	50	20	10	12	28	128.8
Number of usable positions	1’000	1’000	936	937	936	975	1'002.61
Number of segregating sites (*S*)	3.51 (0.62)	4	7	8	6	1	9.22
Number of haplotypes (*k*)	4.41 (0.61)	5	6	6	5	2	10.6
Expected heterozygosity (*h*)	0.65 (0.01)	0.64	0.78	0.89	0.67	0.07	0.755
Nucleotide diversity (*π*) x 10^−3^	0.81 (0.05)	0.77	2.60	2.75	1.74	0.07	1.804
Ewens-Watterson test*^c^*:							
*F*o	0.37 (0.01)	0.38	0.26	0.20	0.39	0.93	0.255
*F*e	0.46 (0.06)	0.44	0.29	0.22	0.30	0.75	0.269
*P*-value	∈ [0.07 ; 0.51]	0.40 (0.93)	0.43 (0.86)	0.48 (0.95)	0.95 (0.83)	**> 0.99 (>0.99)**	1 (0) / 18
Tajima’s *D* test*^d^*:							
*D*	−0.06 (0.36)	−0.30	1.04	−0.11	−0.47	−1.15	0.018
*P*-value	∈ [0.383 ; 0.849]	0.424 (0.935)	0.865 (0.594)	0.483 (0.947)	0.338 (0.637)	0.138 (0.138)	0 / 18
Fu’s *F*_S_ test*^e^*:							
*F*_S_	−0.37 (0.55)	−0.81	0.48	−1.02	−0.55	−1.15	−1.771
*P*-value	∈ [0.245 ; 0.706]	0.312 (0.935)	0.623 (0.863)	0.222 (0.667)	0.318 (0.637)	**0.005 (0.010)**	3 (**1**) / 18

a Average over the 122 samples, standard deviation in brackets.

b Average values for 18 to 20 human populations from four continents; single population values are reported in Supplementary Tables S7 and S12.

c Ewens-Watterson test for departure from selective neutrality and demographic equilibrium; *F*o: observed homozygosity, *F*e: expected homozygosity; the *P*-value is given as the proportion of random *F*e values generated under the neutral equilibrium model that are smaller than, or equal to the observed *F*o value. Significant deviations (*P*-value < 0.025 or > 0.975) are shown in bold, and after correction for multiple testing in brackets; for humans, we report the number of population samples associated with a significant deviation (before slash, and significant after correction for multiple testing in bold and brackets) on the total number of population samples tested (after slash, see Supplementary Table S12).

d Tajima’s *D* test for departure from selective neutrality and demographic equilibrium; the *P*-value is given as the proportion of random *D* values generated under the neutral equilibrium model that are smaller than, or equal to the observed *D* value. Significant deviations (*P*-value < 0.025 or > 0.975) are shown in bold, and after correction for multiple testing in brackets; for humans, we report the number of population samples associated with a significant deviation (before slash, and significant after correction for multiple testing in bold and brackets) on the total number of population samples tested (after slash, see Supplementary Table S12).

e Fu’s *F*_S_ test for departure from selective neutrality and demographic equilibrium; the *P*-value is given as the proportion of random *F*_S_ values generated under the neutral equilibrium model that are smaller than, or equal to the observed *F*_S_ value. Significant deviations (*P*-value < 0.02) are shown in bold, and after correction for multiple testing in brackets; for humans, we report the number of population samples associated with a significant deviation (before slash, and significant after correction for multiple testing in bold and brackets) on the total number of population samples tested (after slash, see Supplementary Table S12).

f Twenty tests out of 122 (16%) indicated significant homozygosity excess (*P*-value > 0.975), thus exceeding by 13 tests the expected proportion of 5% (6.1 out of 122) false positives.

g One hundred and six tests out of 122 (87%) indicated significant deviation from neutral expectation (*P*-value < 0.02), thus exceeding by 103 tests the expected proportion of 2% (2.44 out of 122) false positives.

Similarly to *NAT1*, Western (*P. t. verus*) chimpanzees also have the lowest diversity among the *Pan* genus for *NAT2*, with *h* varying from 0.15 in Western (San Diego sample) to 0.38 in Central (*P. t. troglodytes*) chimpanzees, and *π* from 0.13 in Western to 0.50 in Central chimpanzees (Table 6 and Figure 2). However, in contrast to *NAT1*, a significant departure from the expected diversity under selective neutrality and demographic equilibrium was found with at least one test in all species and sub-species before adjustment for multiple testing, with the exception of Nigeria-Cameroon (*P. t. ellioti*). All observed deviations were either due to an excess of homozygosity (Western-*verus*, Central-*troglodytes* and Eastern-*schweinfurthii* sub-species), and/or to an excess of rare (recent) haplotypes, yielding negative values of Tajima’s *D* (BPRC sample of Western-*verus* chimpanzees, and Eastern-*schweinfurthii* chimpanzees) or Fu’s *F*_S_ (Western chimpanzees and bonobos). For Western chimpanzees, all three tests were significant in the BPRC sample and Fu’s *F*_S_ test remained so after correction for multiple testing. The results were less clear-cut in the 122 sub-samples of the San Diego sample, as deviations from neutrality were mainly observed with Fu’s *F*_S_ test (16%, 0% and 87% significant deviations with the Ewens-Watterson, Tajima’s *D* and Fu’s *F*_S_ tests, respectively). Similarly to *NAT1*, due to small sample size, we consider with caution the Ewens-Watterson tests results for Central (*P. t. troglodytes*) and Eastern (*P. t. schweinfurthii*) chimpanzees (and the Tajima’s *D* test result for the latter), although they were still significant after accounting for type I error rate. Nevertheless, taken all together, these results suggest a possible action of directional (positive or purifying) selection at *NAT2*, at least in Western (*P. t. verus*) chimpanzees.

At *NATP*, bonobos (*P. paniscus*) have a particularly low diversity and Western (*P. t. verus*) chimpanzees have the second lowest diversity among the *Pan* genus. Indeed, expected heterozygosity ranges from 0.07 in bonobos, and 0.64 in Western chimpanzees, to 0.89 in Central (*P. t. troglodytes*) chimpanzees, and nucleotide diversity from 0.07 in bonobos, and 0.77 in Western chimpanzees, to 2.75 in Central chimpanzees (Table 6 and Figure 2). While the null hypothesis of selective neutrality and demographic equilibrium was not rejected for any of the *Pan troglodytes* sub-species, deviations due to a significant excess of homozygotes (Ewens-Watterson test) and of rare alleles (Fu’s *F*_S_ test) were found for bonobos (*P. paniscus*), that remained even after correction for multiple testing. Considering that the locus is a pseudogene, its extremely low diversity in bonobos (only two *NATP* haplotypes observed, one of which with a frequency over 96%, Table 4 and Figure 1) and the associated significant results returned by the neutrality tests are surprising and call for further investigation.

Figure 2 highlights a marked difference in diversity levels between the three *NAT* genes, particularly between *NAT1* and *NAT2*. We found only a marginally significant difference in *Pan* expected heterozygosity (*h*) between the two functional genes, *NAT1* and *NAT2*, after correcting for multiple testing (*P* = 0.039), whereas nucleotide diversity (*π*) is significantly higher at *NAT1* than at *NAT2* (*P* = 0. 0065). Since the comparisons of the two functional paralogs with the pseudogene are influenced by the very low diversity of *NATP* in bonobos (*P. paniscus*), we compared again diversity levels between genes considering only chimpanzee sub-species. In *Pan troglodytes* indeed, nucleotide diversity at *NATP* is significantly higher than at each of the two functional genes (*P* = 0.018 for *NAT1* *vs.* *NATP*, and *P* = 0.018 for *NAT2* *vs.* *NATP*, respectively), and expected heterozygosity at *NATP* is significantly higher than at *NAT2* (*P* = 0.012).

### Comparison of NAT genetic diversity between Pan and humans

The total number of human haplotypes represented in the dataset of human populations analyzed here (Supplementary Table S7) is 21 for *NAT1* (3.5 per sample, on average), 44 for *NAT2* (10.7), and 58 for *NATP* (10.6). No deviation from Hardy-Weinberg equilibrium was found for any of the human samples at any of the *NAT* genes, after correction for multiple testing (Supplementary Table S7).

When compared to chimpanzees and bonobos, humans appear to have about five times less diversity than *Pan* at the homologous *NAT1* gene, while four to nine times more diversity than *Pan* at *NAT2* (Figure 2, Table 6 and Supplementary Table S7). In Figure 2, the documented high level of diversity of human *NAT2* is clearly illustrated by its similarity to *NATP* (Sabbagh *et al.* 2018). Both the Mann-Whitney *U* and Student *t*-tests, adjusted for multiple testing, confirm that expected heterozygosity and nucleotide diversity are significantly higher in *Pan* species and sub-species than in human populations at *NAT1*, and significantly lower at *NAT2* (Supplementary Table S11). By contrast, both expected heterozygosity and nucleotide diversity of human populations at the *NATP* pseudogene fall within the range of those observed in chimpanzees (*P. troglodytes*), whereas both estimates were found to be, as expected, extremely low in bonobos. However, differences in *NATP* diversity levels between humans and the *Pan* species and sub-species were not significant, even before multiple testing adjustment, and despite the extremely low estimates of bonobos (all *P*-values > 0.05).

At *NAT1*, while no significant rejection was observed in *Pan*, selective neutrality and demographic equilibrium are rejected in many human samples (Table 6 and Supplementary Table S12). Indeed, each of the three tests of selective neutrality used rejected the null hypothesis at *NAT1* in at least one population, even after correction for multiple testing. In the Ewens-Watterson homozygosity test, the observed homozygosity (*Fo*) was found always higher than the expected (*Fe*), and significantly so in nine of the 19 tested population samples (eight after correction for multiple testing). Similarly, Tajima’s *D* and *F*_S_ values are significantly negative in 11 (one after correction) and eight (three after correction) population samples, respectively. Conversely, while several tests rejected the null neutral equilibrium model at *NAT2* in *Pan*, no rejection was observed for this gene in human populations with any of the three tests, after correction for multiple testing. At *NATP*, a single rejection in humans was observed with Fu’s *F*_S_ test after correction (in the Dinka of Sudan, Supplementary Table S12). Thus, rejection of the neutral equilibrium model is more consistent at *NAT2* in *Pan* than in humans, whereas it is more consistent at *NAT1* in humans than in *Pan*.

### Comparison with NAT genetic diversity in other hominids

Haplotype inference for gorillas and orangutans could not be achieved with high enough confidence, because several unknown positions in the genotypes retrieved from GAGP overlapped with variants detected in the sequenced samples of this study (Supplementary Tables S3, S4 and S5). Thus, only nucleotide diversity was estimated for gorillas and orangutans (Supplementary Table S13). As shown in Supplementary Figure S5, the relative levels of diversity at the three *NAT* genes differ markedly between the two gorilla species available for this study (Western and Eastern gorillas, *G. gorilla* and *G. beringei*, respectively), and between the two orangutan species (Sumatran and Bornean orangutans, *P. abelii* and *P. pygmaeus*, respectively). The results indicate a level of diversity at *NAT1* similar to that of *Pan* for both species of gorillas, thus also higher than in humans, whereas the highest values among all great apes were observed in orangutans, albeit differing markedly between the two orangutan species (Supplementary File S1). At *NAT2*, Western gorillas and both species of orangutans display a diversity level comparable to that of humans, thus higher than *Pan*, whereas the diversity level of Eastern gorillas is comparable to that of *Pan*. Finally, the highest values of diversity among all great apes, including humans, were observed for the two orangutan and one of the gorilla (*G. gorilla*) species at *NATP*, while no diversity was detected in the sample of the other gorilla species (*G. beringei*). These contrasting results between both the two gorilla and the two orangutan species should however be considered with caution in view of the extremely small sample sizes of Eastern gorillas (*n* = 3) and Bornean orangutans (*n* = 6).

## Discussion

The human acetylation polymorphism, discovered in the early 1950s and shown to be responsible for inter-individual variation in drug biotransformation, is considered as the best-known example of a pharmacogenetic trait (Meyer 2004; Agundez 2008a; McDonagh *et al.* 2014). As reviewed in Sabbagh *et al.* (2018), most of its inter-individual variation, further reflected in inter-population variation, is due to a high number of non-synonymous polymorphisms in the coding region of the *NAT2* gene, which ranks among the most polymorphic human drug metabolizing genes. By contrast, human *NAT1*, its functional paralog located some 200 Kb upstream, displays up to 13 times less diversity than *NAT2* in its coding region. Several observations suggest that *NAT1* and *NAT2* evolved under distinct selective regimes in humans. For human *NAT1*, it is generally held that its product is functionally constrained, and thus subjected to purifying selection, probably because of the expression of the NAT1 enzyme in many tissues early during development (including reproductive tissues), and its implication in the metabolism of folates (Patin *et al.* 2006a; Butcher and Minchin 2012). For human *NAT2* however, due to the role of its product in the detoxification of exogenous substances, it is proposed that the mode of subsistence and/or the chemical environment in which past populations have been living induced positive population-specific selective pressures on the gene, thereby explaining the documented differential distribution of acetylation prevalence among subsistence strategies and exploited biomes (Patin *et al.* 2006a; Luca *et al.* 2008; Magalon *et al.* 2008; Sabbagh *et al.* 2008; Mortensen *et al.* 2011; Podgorná *et al.* 2015). To gain further insights on these evolutionary hypotheses, we performed here a comprehensive analysis of the diversity of *NAT* genes in hominids (*i.e.*, in chimpanzees, gorillas, orangutans, and hominins – humans, including Neanderthal and Denisova). To the best of our knowledge, this is the first study investigating *NAT* intra-species polymorphism in hominids.

### Levels and patterns of diversity of the functional arylamine N-acetyltransferase genes in the Pan genus point to different selective pressures acting on NAT1 and NAT2

Several lines of evidence have led to the current view that, in humans, the diversity of the *NATP* pseudogene can be largely ascribed to our demographic history, but that of *NAT2* was further shaped by population-specific selective pressures, whereas that of *NAT1* was constrained by relatively strong purifying selection (Sabbagh *et al.* 2018). Indeed, a marked geographic structure characterizes human *NATP* diversity, with less diversity in populations from Europe, Asia and the Americas than in African populations (as illustrated in Supplementary Figure S6 for the human dataset assembled in this study). For *NAT2*, the observations are of an excess of non-synonymous relative to synonymous SNPs, a lack of correlation of genetic and geographic distances at the worldwide scale, and an unusual pattern of population structure that differentiates Asian populations from Africans and Europeans more than expected on neutral grounds. Finally, *NAT1* is much less polymorphic in humans, and is associated with a significant excess of homozygotes in many populations.

In *Pan*, the frequency distributions of inferred haplotypes suggest that *NAT* genes have evolved under different selective regimes in these species too. Similarly to humans, these distributions are characterized by marked differences between the three *NAT* genes (Figure 1 and Table 4). At the *NATP* pseudogene, we found extensive variation between *Pan* species and sub-species, and the shapes of these frequency distributions resemble an expected L-shaped neutral distribution (*i.e.*, a haplotype at intermediate to high frequency, other haplotypes at increasingly low frequencies), except in bonobos (*P. paniscus*). At *NAT1*, the commonest haplotype in each subspecies is more frequent than those at *NATP*. The pattern is even more skewed toward a single haplotype at very high frequency at *NAT2*. Moreover, while the same *NAT1* haplotype (Pan NAT1*1) is predominant in each chimpanzee sub-species, at the *NAT2* gene instead different haplotypes are prevalent in each sub-species, except for Eastern (*P. t. schweinfurthii*) and Central (*P. t. troglodytes*) chimpanzees, which is not surprising knowing the related demographic history of these two sub-species (Gagneux *et al.* 1999; Hey 2010; Bjork *et al.* 2011; Prado-Martinez *et al.* 2013; Fünfstück *et al.* 2015; de Manuel *et al.* 2016; Lobon *et al.* 2016).

In line with these observations, our analyses failed to reveal any significant level of genetic differentiation between chimpanzee sub-species at *NAT1*, while we do find significant differentiation at *NAT2*, except between Eastern and Central chimpanzees (Supplementary Table S6). At the *NATP* pseudogene, Western (*P. t. verus*) chimpanzees are significantly differentiated from all the other *P. troglodytes* sub-species (and even between the two *verus* samples), while no genetic differentiation was found among any of the latter. Given the high levels of *NATP* polymorphism in chimpanzees (Figure 1 and Tables 3 and 5) and the significant differentiation between the two *verus* samples, we believe that this lack of genetic differentiation could result from a lack of power due to the small sample sizes available for Central (*P. t. troglodytes*), Eastern (*P. t. schweinfurthii*) and Nigeria-Cameroon (*P. t. ellioti*) chimpanzees. In turn, significant differentiation levels were estimated between bonobos and all other *Pan* at all three *NAT* genes.

In terms of gene and molecular diversity, the different *Pan* species and sub-species display a similar *NAT1* - *NAT2* pattern of variation, *i.e.*, high diversity at *NAT1* and lower at *NAT2*, in spite of differences in diversity levels (Figure 2). In humans instead, the *NAT1* - *NAT2* diversity pattern is reversed, with less diversity than *Pan* at *NAT1* and more at *NAT2*, and these differences between humans and apes are significant (Figure 2 and Supplementary Table S11). Conversely, diversity was not found significantly different between humans and *Pan* at *NATP*. A reversed pattern between *Homo sapiens* and *Pan* was also highlighted by the results of the tests of selective neutrality and demographic equilibrium. While at *NAT1*, only two rejections of the null hypothesis (with one test only) were observed in chimpanzees and bonobos (Table 6), deviations from expectations were found with the three tests in several human populations (Supplementary Table S12). At *NAT2* instead, rejections were observed at least with one test in all chimpanzees and bonobos, except in the *P. t. ellioti* sub-species, while very few rejections were observed in humans (none after correction for multiple testing). Altogether, these results suggest that diversity at *NAT1* in humans could have been influenced by selective pressures (either purifying or recent directional selection), but not in chimpanzees or bonobos, whereas similar selective pressures could have influenced the diversity of the *NAT2* gene in bonobos and at least some chimpanzee sub-species, but not in humans.

However, it is important to consider the possible confounding effects of population demographic history when interpreting any significant departure of nucleotide polymorphism from equilibrium-neutral predictions. Demographic and selective events tend to leave very similar traces over sequences. For example, a selective sweep, *i.e.*, an episode of recent directional selection, and a population bottleneck followed by expansion are both expected to generate a frequency spectrum skewed toward rare alleles. Indeed, genomic comparisons across large numbers of individual hominids has revealed that modern humans are genetically less variable than most other great apes, notably gorillas and orangutans, and most chimpanzee subspecies, although the difference is less pronounced when compared to Western chimpanzees, Eastern lowland gorillas and bonobos (Gagneux *et al.* 1999; Kaessmann *et al.* 2001; Prado-Martinez *et al.* 2013; Xue *et al.* 2015). This pattern is ascribed to the evolutionary history of our species, which is marked by a series of recent demographic expansions, such as the one following the founder event driving modern humans out of Africa (Li *et al.* 2008; Barbujani and Colonna 2010; Veeramah and Hammer 2014). However, human and non-human hominids do not seem to differ in the genome-wide accumulation of loss-of-function mutations and pseudogenes, suggesting that neither human demographic expansions nor the potential buffering role of human culture apparently led to human genomes tolerating a higher mutational load (Prado-Martinez *et al.* 2013). Additionally, according to (Prado-Martinez *et al.* 2013), all chimpanzees and bonobos experienced a population bottleneck more than 2 million years ago, while Western chimpanzees experienced a second, more recent bottleneck some 500,000 years ago, followed by re-expansion. Moreover, studies on the major histocompatibility complex (MHC) region of chimpanzees suggest that a strong selective sweep within this region, owing to the action of a particular viral pathogen, was likely coupled to the first bottleneck (de Groot *et al.* 2002; de Groot *et al.* 2008), and a comparative study of the MHC region in humans and Western chimpanzees suggests that this first bottleneck accounts for observed differences of MHC diversity between the two species. The lower diversity observed in Western chimpanzees compared to the other *P. troglodytes* sub-species (Figure 2 and Table 6) could indeed reflect footprints of the second, more recent bottleneck event, thus implying that the *P. t. verus* sub-species (Western chimpanzees) experienced greater genetic drift than the others. Indeed, it is in Western chimpanzees, among all sub-species of chimpanzees, that the lowest or one of the lowest estimations of diversity was found, including at the *NATP* pseudogene, in agreement with a well-known result at the genome level, *i.e.*, with Central chimpanzees being generally the more diverse and Western chimpanzees the least (Prado-Martinez *et al.* 2013; Bataillon *et al.* 2015; de Manuel *et al.* 2016). Despite these complex demographic histories, the neutrality tests used are known to have no statistical power to detect such ancient demographic events (Tajima 1989b; Tajima 1989a; Simonsen *et al.* 1995; Ramírez-Soriano *et al.* 2008). Consequently, the chimpanzee and bonobo populations investigated can be reasonably assumed to be at demographic equilibrium and null values of Tajima’s *D* and Fu’s *F*_s_ are expected at putatively neutral genomic regions (*e.g.*, non-genic and intronic regions, or pseudogenes). Previous studies investigating such regions have consistently produced null *D* values for Western chimpanzees (Deinard and Kidd 1999; Kaessmann *et al.* 1999; Fischer *et al.* 2004; Fischer *et al.* 2006; Fischer *et al.* 2011; Sugawara *et al.* 2011). Therefore, the significant deviations from the standard, neutral equilibrium model at *NAT2* in chimpanzees and bonobos can be interpreted, in our view, as evidence for some kind of selection, most likely purifying selection, which is how such patterns are interpreted for *NAT1* in humans (Sabbagh *et al.* 2018).

On the other hand, the few rejections of the null hypothesis at *NAT1* in *Pan* rather support a lack of selective pressure at this gene, but this conclusion fails to explain the lack of *NAT1* genetic differentiation among chimpanzees, with the same predominant haplotype in all *P. troglodytes* (*i.e.*, NAT1*1), as opposed to the patterns characterizing *NAT2* and *NATP*. In view of the observed similar level of diversity between humans and chimpanzees at the *NATP* pseudogene, the differences found between the two functional *NAT* paralogs, both among *Pan*, and between *Pan* and humans rather argue for some contribution of selective pressures acting on both these genes. Neither the diversity indices that we estimated nor the selective neutrality tests that we performed account for differences in the functional impact of mutations. Considering such functional effects could help to identify the kind of selective pressures at work, and their likely magnitude.

At the *NAT1* gene, haplotype NAT1*1 is the most frequent in all chimpanzee sub-species, whereas it is NAT1*6 in bonobos (Figure 1). These two haplotypes are nevertheless likely to confer a similar enzymatic profile since they only differ by a synonymous SNP (T369C, Table 3). Therefore, it is expected that a majority of chimpanzees and bonobos have a similar NAT1 acetylation capacity. Moreover, at each of the *NAT1* positions that are polymorphic in *Pan*, all other great apes, including humans are apparently fixed on the nucleotides of the major *Pan* haplotype NAT1*1. Thus, the maintenance of NAT1*1 for approximately half a million years (TMRCA of *Pan troglodytes*) and of a likely similar acetylation capacity (NAT1*6 differs from NAT1*1 by a single synonymous substitution) for about 2 million years (TMRCA of *Pan*) points to the effect of purifying selection acting on this gene to preserve such acetylation capacity. At *NAT2*, as already stated, frequency distributions in *Pan* were found to differ markedly from those at *NAT1*, not only because they are more skewed toward a high frequency of the major haplotype, but also because this prevalent haplotype is different between sub-species. *Pan NAT2* is also characterized by a low level of diversity compared to *NAT1* and *NATP*, and to human *NAT2* as well (Figure 2). Theoretically, such pattern could be due either to rapid genetic drift in populations of chimpanzees and bonobos, or to directional selection, possibly purifying selection, acting to preserve specific haplotypes, depending on the sub-species. Both hypotheses are supported by the tests of selective neutrality and demographic equilibrium, which revealed several rejections in favor of directional selection or a population expansion after a bottleneck. Moreover, if only polymorphic positions within the *Pan* coding exon are considered (Table 3 and Supplementary Figure S2), only three main *NAT2* haplotypes are observed, *i.e.*, NAT2*1/NAT2*4 (most frequent in *P. t. verus*, *P. t. troglodytes* and *P. t. schweinfurthii*), NAT2*6 (*P. t. ellioti*), and NAT2*7 (*P. paniscus*). Thus, although haplotype variation between chimpanzee sub-species is apparently higher at *NAT2* than at *NAT1*, it is likely that the evolution of *NAT2* in *Pan* is nevertheless functionally constrained. This idea finds support in the comparison of *NAT2* with the highly variable *NATP* pseudogene. In spite of this, similarly to observations in humans (Patin *et al.* 2006a; Mortensen *et al.* 2011), significant linkage disequilibrium among the three *NAT* gene family members is mainly detected between *NAT2* and *NATP* (Supplementary Table S14, tested only in Western chimpanzees). It is thus reasonable to assume that the pseudogene has been more free to accumulate recent mutations, while selection acts to remove harmful mutations in *NAT2*. In this context, the extremely low *NATP* diversity in bonobos is intriguing, the more so as their diversity pattern at *NAT1* and *NAT2* is similar to that of chimpanzees (Figure 2). We verified that this depleted polymorphism in the pseudogene could not be ascribed to a lower quality of the SNP calling process or lower coverage for bonobos in the GAGP data. Note that in the GAGP whole genome study, bonobos and also Eastern gorillas (*G. beringei*) showed the lowest genetic diversity among all great apes, and displayed distributions of homozygosity tracts similar to those of human populations having experienced strong genetic bottlenecks (Prado-Martinez *et al.* 2013; Xue *et al.* 2015).

### Prediction of the existence of different profiles of acetylation in chimpanzees and bonobos

In humans, polymorphisms identified in *NAT1* and *NAT2* led to the definition of haplotypes with a known acetylation profile when an association between a mutation and the functionality of the protein was observed *in vivo* or *in vitro* (Walraven *et al.* 2008; Zhu and Hein 2008; Zhu *et al.* 2011). In other primates, for the time being, functional knowledge only exists for the rhesus macaque *NAT2* gene. Indeed, a recent study (Tsirka *et al.* 2014) demonstrated that the function of the NAT2 enzyme in the human and rhesus macaque species diverges in substrate selectivity, shifting substrate affinity of the enzyme between bulkier NAT2 substrates and smaller NAT1 substrates, and that this shift is due to a single substitution, that does not otherwise alter the stability and the overall activity of the protein. Such knowledge does not exist yet for chimpanzees, and we thus used three *in silico* tools to predict the potential consequence of a substitution on the function of *Pan* NAT proteins (Table 5).

We therefore suggest that, compared to the *Pan* basal haplotype NAT1*1, nine derived haplotypes might translate into an equivalent phenotype, whereas two might have a moderate damaging effect. Indeed, none of the SNPs detected in *NAT1* have been predicted as damaging by the three tools concomitantly, but the comparison of our results for *Pan* haplotypes with the outcomes of predictions for human haplotypes with known effects raises the possibility that two *Pan NAT1* haplotypes, NAT1*4 and NAT1*7, could have a moderate “slowing” effect on enzymatic activity (similar to that of human haplotype NAT1*14B, when considering the similarity of the results outputted by the prediction tools).

At the *NAT2* gene, non-synonymous mutations defining three *Pan* derived haplotypes, NAT2*2, NAT2*8, and NAT2*9, were predicted as damaging, with good confidence by the three tools. A slower enzymatic activity associated with these haplotypes could thus be expected. Two of these three haplotypes are rather uncommon, each being observed at a frequency below 5% in one species only: NAT2*2 in Western chimpanzees (*P. t. verus*) and NAT2*8 in bonobos (*P. paniscus*) (Table 4, Figure 1, Supplementary Table S4, and Supplementary Figure S3). Haplotype NAT2*9 was also detected in bonobos only, but it has an estimated frequency over 7%. Hence the cumulated frequencies of *NAT2* haplotypes potentially conferring slower NAT2 enzymatic activity in *P. paniscus* could reach 10%. This translates into an expected frequency of carriers of two potentially slow haplotypes of 1%, *vs.* 18% of heterozygous slow/rapid carriers (observed frequencies were of 0% and 21.4%, respectively). By comparison, the lowest frequencies of NAT2 slow acetylators in human populations (either directly documented by phenotypic studies, or indirectly estimated by the proportion of carriers of two slow haplotypes) vary by around 10% (Sabbagh *et al.* 2011). Such low frequencies are encountered both in hunter-gatherer populations around the world (with the lowest values among some hunter-gatherer populations of the American continent (Fuselli *et al.* 2007), and in some North East Asian populations (in China, Korea and Japan). For the *Pan* species, we tentatively speculate that two additional haplotypes (NAT2*6 and NAT2*7) could be associated with a substrate-dependent reduction in acetylation activity, given the similarity of results returned by the prediction tools with those of human haplotypes for which such substrate-dependent activity has been proposed (*i.e.*, human NAT2*7A, NAT2*7B, and NAT2*10, Supplementary File S1 and Supplementary Table S15). Although we acknowledge that such assertion is based on very limited evidence, it nevertheless opens up the possibility that, compared to chimpanzees, NAT2 activity in bonobos could be globally reduced given that NAT2*7 is the most prevalent haplotype in this species (Figure 1 and Supplementary Figure S3).

In summary, in the present state of knowledge, our results suggest the existence among chimpanzees and bonobos, as in humans, of diversified acetylation profiles for both *NAT1* and *NAT2* genes. While for *NAT1* only two infrequent *Pan* haplotypes might confer a slower enzymatic activity than the reference, for *NAT2*, two of the three haplotypes observed in bonobos are predicted to do so, as well as one in chimpanzees. In terms of frequencies, however, the majority of *NAT2* haplotypes observed in chimpanzees are likely associated with a “normal” acetylation capacity that would be the *Pan* equivalent corresponding to human rapid acetylation (Supplementary File S1 and Supplementary Table S16). It is thus likely that, in chimpanzees, mutations modifying the functionality of the NAT1 and NAT2 enzymes in a fashion that slows acetylation could be subject to purifying selection. Purifying selection acting on the chimpanzee *NAT2* gene is consistent with its low diversity within sub-species as compared to *NATP*, and its high molecular differentiation among *Pan* sub-species. However, the greater molecular diversity found at *NAT1* within sub-species is more compatible with a mechanism of (weak) positive directional selection favoring the prevalent P. troglodytes NAT1*1 haplotype in all sub-species. Although constrained by the sample sizes available, our results raise the possibility that in bonobos, however, functional constraints at *NAT2* against slower acetylation could be less stringent than in chimpanzees, allowing a predicted frequency of NAT2 slow acetylation haplotypes of 10% in this species.

These evolutionary hypotheses need to be tested with functional studies of the *in vitro* and/or *in vivo* activities of NAT1 and NAT2 enzymes in *Pan* species. Moreover, such hypotheses will also need to address further complexities, such as the NAT2 metabolic adaptations (*i.e.*, shifts in NAT2 enzymatic activity) recently reported in a study comparing a local human population with a population of first-generation emigrants (Aklillu *et al.* 2018). Finally, we acknowledge that our analyses are based on only a few segregating sites, which is expected given the short length of the *NATs* open-reading frames. Hence larger sample sizes than the ones available in this study are required to make robust assertions on the prevalence of distinct acetylator profiles, and to confirm the patterns of molecular diversity of *NAT* genes found in chimpanzees and bonobos.

### Divergent selective pressures acting on the evolution of NAT genes in humans and chimpanzees

The results of the three tests of selective neutrality used on the human dataset analyzed here support the current view that human *NAT1* diversity is constrained by purifying selection (Table 6 and Supplementary Table S12). On the other hand, the finding of higher frequencies of human NAT2 slow acetylator phenotypes in food-producing populations compared to hunter-gatherers supports the idea that the gene was impacted by selective pressures induced by the modes of subsistence adopted by past populations, and in particular their diets (Patin *et al.* 2006a; Luca *et al.* 2008; Magalon *et al.* 2008; Sabbagh *et al.* 2008; Mortensen *et al.* 2011; Podgorná *et al.* 2015). However, this selective hypothesis fails to be consistently supported, with tests of selective neutrality on human *NAT2* variation producing very few significant results (including those from this study, although see (Patillon *et al.* 2014)). Several explanations for the observed weak and inconstant signals of selection have been suggested, such as the action of adaptive mechanisms difficult to detect through the standard frequency spectrum tests that we and others have used (*i.e.*, selection on standing variation, selection favoring heterozygotes carrying a slow and a rapid haplotype, ancient balancing selection masked by directional selection on specific haplotypes, or recent relaxation of functional constraints). Moreover, consistent evidence for genetic and genomic signatures of demographic expansions in humans in the past (Li *et al.* 2008; Barbujani and Colonna 2010; Veeramah and Hammer 2014) could have mitigated molecular signals of selective mechanisms such as balancing selection and/or selection on standing variation. Interestingly, in human populations from the African Sahel and surrounding regions, higher proportions of *NAT2* rapid acetylators were found not only among hunter-gatherers, as opposed to food-producers, but also among populations living in humid tropical environments, as opposed to those living in more arid zones, independently of their mode of subsistence. It thus raises the possibility that selective pressures on *NAT2* could be exerted not only by shifts to new dietary habits, but by the natural chemical environment as well (Podgorná *et al.* 2015).

Our results suggest that the diversity of *NAT* genes in chimpanzees could result from evolutionary forces that differ from those operating in humans. Today, chimpanzees live mainly in humid tropical environments (according to the classification of the United Nations Environment Program) even if the limits of their habitat are also located in more arid zones (such as in Senegal, Guinea, Mali, Ivory Coast, Uganda, Tanzania and the Republic of Congo). Besides the differences in the overall efficiency of purifying and positive selection acting on the genomes of great ape species, which was shown to be correlated with their long-term population sizes (Cagan *et al.* 2016), differences in intensity of selective pressures exerted by the environment could also be invoked. Chimpanzees and bonobos are raw food “hunter-gatherers” that feed mainly on plant matter, but also eat uncooked insects, birds, eggs and small- to middle-sized mammals. Moreover, it is generally assumed that chimpanzee and bonobo diets have not substantially changed over time, in contrast to humans, whose diets have done so, probably several times, over the last 200 thousand years.

In humans, besides their influence on the effectiveness of prescribed medications, polymorphisms at *NAT2*, and also at *NAT1*, have been associated with differential susceptibility to various cancers linked to arylamine exposure (Hein 2002; Agundez 2008b; Ladero 2008; Selinski *et al.* 2015; Hein 2018; Laurieri *et al.* 2018). Such exposures occur with cigarette smoke, gases and pollutants produced by various chemical industries, as well as diets including meat or fish cooked (or fried) at high temperatures (Weisburger 2002; Chung 2015; Fahrer 2016). Patin *et al.* (2006a) stressed the idea that changes in exposure to xenobiotics with carcinogenic risk or other toxicities associated with the new diets introduced by the transition to food-producing life-styles, as opposed to hunter-gatherer subsistence modes, might have led to changes in the selective pressures acting on drug-metabolizing enzymes such as the NAT enzymes. Here we speculate that the entire phenomenon of food processing, which is intimately linked to the handling of fire, and thus represents a major distinctive human feature common to all modes of subsistence, may have exposed our species to new, food-borne carcinogens and other toxic molecules seldom encountered in the diets of other primate species. Human biological adaptation stemming from the controlled use of fire is an idea that runs back at least to Charles Darwin (Wrangham and Carmody 2010). It is supported, notably, by studies on the influences of a cooked diet on gene expression in the liver (Carmody *et al.* 2016), and the hypothesis of a specific genetic adaptation to fire use is advocated to explain the fixation, in modern humans, of a single nucleotide substitution in the aryl hydrocarbon receptor (*AHR*) gene, which results in a lowered sensitivity of the receptor to toxic exogenous AHR-ligands, such as polycyclic aromatic hydrocarbons (PAHs) that are contained in fire smoke and cooked and smoked foods (Hubbard *et al.* 2016).

Turning back to chimpanzees, we could thus speculate that living in a more limited environment and having experienced little changes in diet, selective pressures such as those affecting humans have been less intense or even non-existent. Instead, the hypothesis of purifying selection acting to maintain an acetylation activity sufficiently adapted to the chimpanzees’ environment and diet is consistent with their low diversity at *NAT2*, the significant rejections of neutrality for this gene, at least for Western chimpanzees, and the low frequencies of those mutations that were predicted to be damaging. While *NAT2* mutations leading to a slower acetylation phenotype are hypothesized to have recently become advantageous in many human populations as they settled in new environments and/or adopted new subsistence strategies, including the consumption of cooked and roasted foods, such mutations are likely to have been deleterious in chimpanzees, and thus negatively selected.

Although it is likely that chimpanzees and bonobos did not experience similar shifts in diets as humans did, the hypothesis of a less stable chemical environment for humans than for other great apes is challenged by major climatic changes, such as the drier conditions developing in the Last Glacial Maximum which have been proposed to be associated with the speciation process leading to Eastern and Western gorillas, and the demographic decline of the latter species (Roy *et al.* 2014; Xue *et al.* 2015). Moreover, our analyses did not produce systematically matching results between Western chimpanzees and the other sub-species, in particular with regards to the selective neutrality tests. *A priori*, these discrepancies could result from the smaller sample sizes of Central, Eastern and Nigeria-Cameroon chimpanzees, making miss-estimation of haplotype frequencies a possible issue. Note that the sample sizes of Western chimpanzees (18 and 23 individuals for San Diego and BPRC, respectively, Table 4) are smaller than the average those of the human populations samples analyzed here (between 60 and 70 individuals, Supplementary Table S7), although several human samples from African populations are represented by less than 20 individuals. Moreover, one cannot exclude the possibility of sequencing errors, particularly for those haplotypes defined by singleton SNPs and observed only once (*e.g.*, Eastern chimpanzee Andromeda, whose data were retrieved from the GAGP, is the only carrier of NAT2*10). Finally, sub-species determination, very often exclusively based on mitochondrial DNA, could be erroneous, especially so in the presence of hybrids (Becquet *et al.* 2007); see also Supplementary File S1).

Sample sizes at least comparable to those of Western chimpanzees in the present study are thus needed to confirm the apparent differences between sub-species that we detected. The recent publication of new genomes for these sub-species (de Manuel *et al.* 2016) is likely to allow an evaluation of our findings in the near future. These findings also call for studies on chimpanzees living in different environments and under different chemical exposure. For instance, numerous Eastern chimpanzees of the Sebitoli community present congenital anomalies as well as palate clefts (Krief *et al.* 2014; Krief *et al.* 2015; Krief *et al.* 2017). In humans, deficiency in folates, in which NAT1 is implicated, is often associated with many congenital malformations including palate clefts (Wahl *et al.* 2015), and some mutations in both *NAT1* and *NAT2* have been associated with this condition (Song *et al.* 2013; Santos *et al.* 2015).

### A hypothetical shift in function between NAT1 and NAT2 during hominid evolution

The differences in diversity levels between *NAT1* and *NAT2* among great ape species suggest that, as in humans, a differentiated function for the two enzymes exists also in other hominid species. In humans, the NAT1 and NAT2 isoenzymes have a different expression profile and acetylate different substrates (Jensen *et al.* 2006; Wakefield *et al.* 2007; Butcher and Minchin 2012; Laurieri *et al.* 2014; Sim *et al.* 2014). Human NAT1 is expressed in most tissues from very early during development and is assumed to play a role in the metabolism of folates, while NAT2 is mostly expressed in the liver and intestines and its substrates are not supposed to influence its regulation, contrarily to NAT1. As reviewed by (Butcher and Minchin 2012), the transcriptional and post-transcriptional regulation of the NAT1 enzyme could also have a higher effect on NAT1 activity than the genotype, contrarily to what is known for NAT2. Notably, it is suggested that epigenetic regulation depending on the concentration of some substrates could affect the activity of NAT1 in cells (Wakefield *et al.* 2010). If differences in diversity levels between the two genes are linked to differences in factors contributing to the inter-individual variation in enzymatic activity between NAT1 (high variation in expression regulation) and NAT2 (high variation in protein sequence) is an open question. It would be tempting to assume that the function of the two enzymes is different in chimpanzees, compared to humans, in view of the reversed *NAT1-NAT2* diversity pattern of chimpanzees and other great ape species (bonobos and Sumatran orangutans). For instance, one could consider the possibility of a shift in substrate affinity between the two isoenzymes during hominid evolution, as suggested by the expression study of the rhesus macaque *NAT2* gene (Tsirka *et al.* 2014), leading to a divergence in function between *Pan* and *Homo*. At present, however, neither temporal (*i.e.*, early or later in development), nor spatial (*e.g.*, ubiquitous *vs.* tissue-specific) differences in expression of the two NAT isoenzymes are known in other great ape species besides humans, so we can only speculate on possible environmental factors that could exert a selective pressure on *NAT* genes in our closer relatives.

In conclusion, we have found high levels of diversity of *NAT* genes in chimpanzees and bonobos, which is similar to humans. However the diversity is reversely distributed in chimpanzees and bonobos, such that there is higher diversity in *Pan NAT1* and lower diversity in *Pan NAT2*, whereas the opposite is observed in humans. A reversed pattern between *Pan* and humans was also returned by the tests of selective neutrality and demographic equilibrium. Rejections of the model in chimpanzees were found mostly associated with *NAT2*, and likely due to directional selection, whereas in human populations this appears to be the case for *NAT1*, thus suggesting distinct selective pressures acting on *Pan NAT1* and *NAT2* compared to humans. Our analyses of the predicted functional impact of mutations detected in non-human primates suggest that a non-negligible proportion of chimpanzees could have a moderately reduced *NAT1* acetylation capacity, in sharp contrast with most human populations. In turn, reduced *NAT2* acetylation capacity is known to be frequent in many human populations, and our analyses predicted that this could also be the case for a significant proportion of bonobos, but less so of chimpanzees. Altogether, our results raise the possibility that humans and chimpanzees evolved some divergence in functionality at the *NAT* genes in the course of hominid history, such as divergence in substrate affinity/specificity/selectivity for each of the two enzymes. Such hypothetical shift in function could be due to fixed substitutions between humans and *Pan NAT* genes, as has been shown for macaques compared to humans. On the basis of the known role of *NAT2* in the metabolism of smoke-contained aromatic amines, we postulate that a functional divergence of *NATs* between *Pan* and humans could have been driven by the development of fire handling and food processing in humans, a hypothesis that could be addressed in the future by functional studies of NAT1 and NAT2 enzymatic activities in great apes.
